# Supplementary material for: Protocol of mixed-methods assessment of demographic, epidemiological and clinical profile of decentralised patients with cancer at Nelson Mandela Academic Hospital and Rob Ferreira Hospital, South Africa
Source: BMJ Open. 2022 Apr 21;12(4):e054983. doi: 10.1136/bmjopen-2021-054983 (PMC9024264; doi:10.1136/bmjopen-2021-054983)
Supplement: Supplementary data [file bmjopen-2021-054983supp001.pdf]

Appendix A

Survey questionnaire (patients)

INSTRUCTIONS: Fill in the blank spaces with a tick where appropriate.

Date of Administration: ____________________________

Section 1: demographic profile

1. Gender

1	Female	
2	Male	

2. Date of Birth

Dd/Mm/Yy:

3. Ethnicity

1	African	
2	White	
3	Indian	
4	Coloured	
5	Other: specify	

4. Marital status

1	Never Married	
2	Married (including lobola)	
3	Divorced/Separated	
4	Widowed	
5	Cohabiting	

5. What is the highest standard/grade or level you have passed at school or tertiary education?

Chitha W, *et al. BMJ Open* 2022; 12:e054983. doi: 10.1136/bmjopen-2021-054983

6. Are you currently studying?

1	Yes	
2	No	

7. What is your current level of study? --

8. Are you employed?

a)

1	Yes	
2	No	

b) If employed, what type of work do you do? --

9. Source of income (tick all appropriate)

1	2	3	4	5	6	7	8	9
Job	Old Age Grant	Disability Grant	Other Pension	Spousal support	Support from children	Child support grant	None	Other (Specify)

10. What is your gross family income each month (that is, before tax)?

1	No income		7	R6 401 – R12 800	
2	R1 – R400		8	R12 801 – R25 600	
3	R401 – R800		9	R25 601 – R51 200	
4	R801 – R1 600		10	R51 201 – R102 400	
5	R1 601 – R3 200		11	R102 401 – R204 800	
6	R3 201 – R6 400		12	More than R204 800	

11. What is the name of your place of residence?____

12. Which health facility referred you here (Name)?

____

Section 2: Epidemiological and clinical profile of various cancers

13a. Do you have a family history of cancer?

1	2	3
Yes	No /	Unsure

13b. If yes to above, how are you related to the family member(s) who has the cancer diagnosis?

____
____

13c. What type of cancer(s) does the family member(s) have?

____

____

____

14. Do you smoke? (tick all appropriate)

a)

1	Yes	
2	No	

b) If Yes, when did you start smoking?

Year: __________

c) On average, how many cigarettes do you smoke in a day? ____________________

d) If No, have you ever smoked?

1	Yes	
2	No	

e) For how long did you smoke? __________________

f) How many did you smoke in a day? ________________

1	Once	
2	Twice	
3	Three time	
4	More than 3 times	

Chitha W, *et al. BMJ Open* 2022; 12:e054983. doi: 10.1136/bmjopen-2021-054983

g) Did you stop smoking?

1	Yes	
2	No	

h) How long ago did you stop smoking? ______________________________________

15. Do you drink alcohol?

a)

1	Yes	
2	No	

b) Did you drink alcohol before?

1	Yes	
2	No	

16. Do you exercise (physical) on a regular basis?

1	Yes	
2	No	

17. Have you ever worked in mines?

1	Yes	
2	No	

18. How long in years did you work in mines? --

19. Which mines (tick all that apply)? --

1	Gold	
2	Platinum	
3	diamond	
4	Coal	
5	Other (specify)	

Chitha W, *et al. BMJ Open* 2022; 12:e054983. doi: 10.1136/bmjopen-2021-054983

20. Please indicate if your family has a history of any cancer/s below?

1	Breast cancer		6	Uterus cancer	
2	Lung cancer		7	Colon cancer	
3	Cervical cancer		8	Ovarian cancer	
4	Prostate Cancer		9	Other (specify)	
5	Oesophagus cancer		10	No history of cancer in my family	

21. Before you were told you needed to go to hospital about cancer, how many times did you see other doctors or health professionals about the health problem caused by cancer?

		1x	2x	3x	4x	5x	Other
1	I visited my local clinic						
2	I visited my local hospital						
3	I saw my local private doctor						
4	I saw my traditional healer/doctor/ Isangoma						
5	Other (specify):						

22. How do you feel about the length of time you had to wait before your first appointment with a hospital doctor or clinic doctor?

1	I was seen as soon as I thought was necessary	
2	I should have been seen a bit sooner	

23. How long was it from the time you identified symptoms?

----

24. Did your symptoms get better or worse or were the same while you were waiting for your first appointment with a hospital doctor?

----

25. What type of cancer(s) were you diagnosed with?

----

26. When was your cancer(s) diagnosed?

Chitha W, *et al. BMJ Open* 2022; 12:e054983. doi: 10.1136/bmjopen-2021-054983

27. What health problems or symptoms did you notice at first?

----

28. Who first told you that you had cancer?

1	A hospital doctor	
2	A hospital nurse	
3	A GP (family doctor)	
4	Another health professional (specify)____________	
5	A friend or relative	
6	Nobody – I worked it out for myself	
7	Cannot remember	

29. When you were first told that you had cancer, had you been told you could bring a family member or friend with you?

1	Yes	
2	No	
3	It was not necessary	
4	I was told by phone or letter	
5	Don’t know / Can’t remember	
6	Other (specify)_________________	

30. How do you feel about the way you were told you had cancer?

----

30. Did you understand the explanation of what was found with you?

1	Yes	
2	No	

31. When you were told you had cancer, were you given written information about the type of cancer you had?

1	Yes	
2	No	

Chitha W, *et al. BMJ Open* 2022; 12:e054983. doi: 10.1136/bmjopen-2021-054983

32. Before your cancer treatment started, were you given a choice of different types of treatment?

1	Yes	
2	No, but I would have liked a choice	
3	I was not given a choice because only one type of treatment was suitable for me	
4	Not sure / Can't remember	
5	Missing	

33. Do you think your views were taken into account when the team of doctors and nurses caring for you were discussing which treatment you should have?

1	Yes	
2	No	

34. Were the possible side effects of treatment(s) explained in a way you could understand?

1	Yes	
2	No	

35. Before you started your treatment, were you given verbal/written information about the side effects of treatment(s)?

1	Yes	
2	No	

36. Were you involved as much as you wanted to be in decisions about your care and treatment?

1	Yes	
2	No	

37a. During the last 12 months, have you had an operation (such as removal of a tumour or lump) at one of the hospitals named in the covering letter?

1	Yes	
2	No	
3	Not sure	

b. Before you had your operation, did a member of staff explain what would be done during the operation?

1	Yes	
2	No	

38. The last time you went into hospital for a cancer operation, was your admission date changed to a later date by the hospital?

1	Yes	
2	No	

39. Beforehand, were you given written/verbal information about your operation?

1	Yes	
2	No	

40. After the operation, did a member of staff explain how it had gone in a way you could understand?

1	Yes	
2	No	

41. As far as you know, was the hospital or your doctor that referred you for cancer treatment given enough information about your condition and the treatment you had at the hospital?

1	Yes	
2	No	
3	Don’t know / Can’t remember	

42. Do you think the doctors and nurses at your local hospital or clinic did everything they could to support you while you were in their care?

1	Yes	
2	No	

Chitha W, *et al. BMJ Open* 2022; 12:e054983. doi: 10.1136/bmjopen-2021-054983
