# Supplementary material for: Protocol of mixed-methods assessment of demographic, epidemiological and clinical profile of decentralised patients with cancer at Nelson Mandela Academic Hospital and Rob Ferreira Hospital, South Africa
Source: BMJ Open. 2022 Apr 21;12(4):e054983. doi: 10.1136/bmjopen-2021-054983 (PMC9024264; doi:10.1136/bmjopen-2021-054983)
Supplement: Supplementary data [file bmjopen-2021-054983supp002.pdf]

Appendix C: Questionnaire validation

	Relevance						Clarity					
	Expert _1	Expert _2	Expert _3	Experts in agreement	Item Content Validity Index (I-CVI)	Universal Agreement (UA)	Expert _1	Expert _2	Expert _3	Experts in agreement	Item Content Validity Index (I-CVI)	Universal Agreement (UA)
**Survey questionnaire (patients) INSTRUCTIONS: Fill in the blank spaces with a tick where appropriate. Date of Administration: ______________**												
**Section 1: demographic profile**												
**Question**												
1. Gender 1 Female 2 Male	4	4	4	3	1	1	4	3	4	3	1	1
2. Date of Birth Dd/Mm/Yy: ______________	4	3	4	3	1	1	4	4	4	3	1	1
3. Ethnicity 1 African 2 White 3 Indian 4 Coloured 5 Other: specify	4	4	4	3	1	1	4	4	4	3	1	1
4. Marital status 1 Never Married 2 Married 3 Divorced/ Separated 4 Widowed 5 Cohabiting	3	3	4	3	1	1	4	3	4	3	1	1
5. What is the highest standard/grade you have attended in education? ------------	4	4	3	3	1	1	4	3	4	3	1	1
6. Are you currently studying? 1 Yes 2 No	3	3	3	3	1	1	4	3	3	3	1	1
7. What is your current level of study? ---------------	3	3	3	3	1	1	4	3	4	3	1	1
8. Are you employed? a) 1 Yes 2 No b) If employed, what type of employment? ----------------------	4	4	4	3	1	1	4	4	4	3	1	1

9. Source of income (tick all appropriate)<br>1 Job<br>2 Old Age Grant<br>3 Disability Grant<br>4 Other Pension<br>5 Spousal support<br>6 Support from children<br>7 Child support grant<br>8 None<br>9 Other	4	4	4	3	1	1	4	4	4	3	1	1
10. What is your residential area? Name of town/administrative area: ---------	3	4	3	3	1	1	4	3	3	3	1	1
11. Referring facility:<br>1 Clinic<br>2 Community Health Centre<br>3 District Hospital<br>4 Regional Hospital<br>5 Private General practitioner<br>6 Private hospital<br>7 Other (specify):	4	4	4	3	1	1	4	4	4	3	1	1
**Section 2: Epidemiological and clinical profile of various cancers**				3	1	1				3	1	1
1. Do you have a family history of cancer?<br>1 Yes<br>2 No<br>3 Unsure	4	4	4	3	1	1	4	4	4	3	1	1
2. Do you smoke? (tick all appropriate)<br>1 Yes<br>2 No	4	4	4	3	1	1	4	3	4	3	1	1
2b) If Yes, when did you start smoking? Year: ___________	4	3	4	3	1	1	4	3	3	3	1	1
2c) On average, how many cigarettes do you smoke in a day?	4	3	4	3	1	1	4	3	4	3	1	1
2d) If No, have you ever smoked?	4	3	4	3	1	1	4	3	4	3	1	1
2e) For how long did you smoke?	4	3	4	3	1	1	4	4	4	3	1	1

2f) How many did you smoke in a day?<br>1 Once<br>2 Twice<br>3 Three time<br>4 More than 3 times	4	3	4	3	1	1	4	3	3	3	1	1
2g) Did you stop smoking?<br>1 Yes<br>2 No	4	3	3	3	1	1	4	4	3	3	1	1
3. Do you drink alcohol?<br>a)<br>1 Yes<br>2 No	4	4	4	3	1	1	4	4	4	3	1	1
4. Do you exercise (physical) on a regular basis?<br>1 Yes<br>2 No	4	4	3	3	1	1	4	4	4	3	1	1
4b) Did you drink alcohol before?<br>1 Yes<br>2 No	4	4	4	3	1	1	4	4	4	3	1	1
5. Have you ever worked in mines?<br>1 Yes<br>2 No	4	4	4	3	1	1	4	3	4	3	1	1
6. How long in years did you work in mines? ------------	4	4	4	3	1	1	4	4	4	3	1	1
7. Which mines? Gold, diamond coal, mixed? --------	4	4	4	3	1	1	4	4	4	3	1	1
8.<br>a) Please indicate if your family has a history of any cancer/s below?<br>1 Breast cancer<br>5 Oesophagus cancer<br>2 Lung cancer<br>6 Colon cancer<br>3 Cervical cancer<br>7 Ovarian cancer<br>4 Prostate Cancer<br>8 Other (specify)<br>9 No history of cancer in my family<br><br>b) If you have history of cancer in your family, indicate who in the family had these cancer(s)?	4	4	4	3	1	1	4	4	4	3	1	1

9. Before you were told you needed to go to hospital about cancer, how many times did you see your GP (family doctor)/ clinic about the health problem caused by cancer?<br>1x 2x 3x 4x 5x Other<br>1 I visited my local clinic<br>2 I saw my local private doctor<br>3 I saw my traditional healer/doctor/ Isangoma<br>4 Other (specify):	4	4	4	3	1	1	4	4	4	3	1	1
10. How do you feel about the length of time you had to wait before your first appointment with a hospital doctor or clinic doctor?<br>1 I was seen as soon as I thought was necessary<br>2 I should have been seen a bit sooner	4	2	4	2	0,67	0	4	3	4	3	1	1
11. How long was it from the time you identified symptoms? ------------------------	4	4	4	3	1	1	3	4	3	3	1	1
12. Did your symptoms get better or worse or were the same while you were waiting for your first appointment with a hospital doctor? ----	4	4	4	3	1	1	4	4	4	3	1	1
13. What type of cancer(s) were you diagnosed with? ---	4	4	4	3	1	1	4	4	4	3	1	1
14. When was your cancer(s) diagnosed? ---------	4	4	4	3	1	1	4	4	4	3	1	1
15. What health problems or symptoms did you notice at first? ------	4	4	4	3	1	1	4	3	4	3	1	1

16. Who first told you that you had cancer?<br>1 A hospital doctor<br>2 A hospital nurse<br>3 A GP (family doctor)<br>4 Another health professional<br>5 A friend or relative<br>6 Nobody – I worked it out for myself<br>7 Cannot remember	3	4	4	3	1	1	4	4	4	3	1	1
17. When you were first told that you had cancer, had you been told you could bring a family member or friend with you?<br>1 Yes<br>2 No<br>3 It was not necessary<br>4 I was told by phone or letter<br>5 Don't know / Can't remember<br>6<br>Missing	3	4	4	3	1	1	3	4	4	3	1	1
18. How do you feel about the way you were told you had cancer? --------	4	4	4	3	1	1	4	4	4	3	1	1
19. Did you understand the explanation of what was found with you? --------	4	4	4	3	1	1	4	4	4	3	1	1
20. When you were told you had cancer, were you given written information about the type of cancer you had? -----	4	4	4	3	1	1	4	4	4	3	1	1
21. Before your cancer treatment started, were you given a choice of different types of treatment?<br>1 Yes<br>2 No, but I would have liked a choice<br>3 I was not given a choice because only one type of treatment was suitable for me<br>4 Not sure / Can't remember<br>5 Missing	4	4	4	3	1	1	4	4	4	3	1	1

22. Do you think your views were taken into account when the team of doctors and nurses caring for you were discussing which treatment you should have? ------	4	4	4	3	1	1	4	4	4	3	1	1
23. Were the possible side effects of treatment(s) explained in a way you could understand? --------	4	4	4	3	1	1	4	4	4	3	1	1
24. Before you started your treatment, were you given verbal/written information about the side effects of treatment(s)? -------	4	4	4	3	1	1	4	4	3	3	1	1
25. Were you involved as much as you wanted to be in decisions about your care and treatment? -----	4	4	4	3	1	1	4	4	4	3	1	1
26. a) During the last 12 months, have you had an operation (such as removal of a tumour or lump) at one of the hospitals named in the covering letter?<br>1 Yes<br>2 No<br>3 Not sure	4	4	4	3	1	1	4	4	4	3	1	1
26.b) Before you had your operation, did a member of staff explain what would be done during the operation? --------	4	4	4	3	1	1		4	4	3	1	1
27. The last time you went into hospital for a cancer operation, was your admission date changed to a later date by the hospital? -----------	4	4	4	3	1	1	4	4	4	3	1	1
28. Beforehand, were you given written/verbal information about your operation? ---	4	4	4	3	1	1	3	4	4	3	1	1

29. After the operation, did a member of staff explain how it had gone in a way you could understand? -------------	4	4	4	3	1	1	4	4	4	3	1	1
30. As far as you know, was your doctor given enough information about your condition and the treatment you had at the hospital?<br>1 Yes<br>2 No<br>3 Don't know / Can't remember	4	3	4	3	1	1	3	3	4	3	1	1
31. Do you think the doctors and nurses at your general practice/local clinic did everything they could to support you while you were at general practice or local clinic? ------	4	4	4	3	1	1	4	3	4	3	1	1
	**Total**				**50,67**	**50**					**51**	**51**
	Average Score-Content Validy Index (**S-CVI**) = I-CVI/n				**0,99352941**	**0,98**				**S-CVI**	**1**	**1**
	n = 51											
	**Proportion relevance**						**Proportion Clarity**					
	Expert 1	1					Expert 1	1				
	Expert 2	0,98					Expert 2	1				
	Expert 3	1					Expert 3	1				

**Likert Scale:**	
**Relevance**	**Clarity**
1 = Item is not relevant to the measured domain	1 = Item is not clear
2 = Item is somewhat relevant to the measured domain	2 = Item needs some revision
3 = Item is quiet relevant to the measured domain	3 = Item is clear but need some minor revision
4 = Item is highly relevant to the measured domain	4 = Item is very clea
