# Supplementary material for: Protocol of mixed-methods assessment of demographic, epidemiological and clinical profile of decentralised patients with cancer at Nelson Mandela Academic Hospital and Rob Ferreira Hospital, South Africa
Source: BMJ Open. 2022 Apr 21;12(4):e054983. doi: 10.1136/bmjopen-2021-054983 (PMC9024264; doi:10.1136/bmjopen-2021-054983)
Supplement: Supplementary data [file bmjopen-2021-054983supp003.pdf]

Appendix B

Document review template

##	Date of birth	Province	Site	Child	Patient Classification	Referred patient	Referral Type	Internal Referral Department	Referring Hospital_ Mpumalanga	---
1.										
2.										
3.										
4.										
5.										

##	Referring Hospital_EC	Referral Hospital	Gender	Race	Citizenship	Medical Aid	Postal code	Employed	Source of income	Specify Occupation	---
6.											
7.											
8.											
9.											
10.											

##	Previous work in mine	Number of Years worked in mines	Marital Status	Date of 1st oncology visit	Date of diagnosis	Cancer diagnosis 1	ICD10_Cancer diagnosis1	Cancer diagnosis1_ Stage	Cancer diagnosis 2	ICD10_Cancer diagnosis2	---
11.											
12.											
13.											
14.											
15.											

##	Cancer diagnosis2_Stage	Cancer diagnosis3	ICD10_Cancer diagnosis3	Cancer diagnosis3_ Stage	Chemotherapy	Onco_Drug1	Onco_Drug2	Neupogen	Hormonal Therapy	Blood Transfusion	---
16.											
17.											
18.											
19.											
20.											

##	Radiotherapy	PET_Bone Scan	Palliative Care	Social support	Psychological support	HIV	Hypertension	Diabetes Mellitus	COPD	Asthma	---
21.											
22.											
23.											
24.											
25.											

##	Other Chronic Disease1_Name	Other Chronic Disease2_Name	Family history of cancer	Previous Smoker	Current Smoker	Number of years of smoking	Number of cigarettes smoked in a day	Weight in Kg	Height in Centimetres	Date_of_Current Visit1	---
26.											
27.											
28.											
29.											
30.											

##	Follow-up Date1	RIP Date	Date_of_Current Visit2	Follow-up Date2	---
31.					
32.					
33.					
34.					
35.					

Chitha W, *et al. BMJ Open* 2022; 12:e054983. doi: 10.1136/bmjopen-2021-054983
