# Supplementary material for: Protocol of mixed-methods assessment of demographic, epidemiological and clinical profile of decentralised patients with cancer at Nelson Mandela Academic Hospital and Rob Ferreira Hospital, South Africa
Source: BMJ Open. 2022 Apr 21;12(4):e054983. doi: 10.1136/bmjopen-2021-054983 (PMC9024264; doi:10.1136/bmjopen-2021-054983)
Supplement: Author's manuscript [file bmjopen-2021-054983.draft_revisions.pdf]

BMJ Open

BMJ Open is committed to open peer review. As part of this commitment we make the peer review history of every article we publish publicly available.

When an article is published we post the peer reviewers' comments and the authors' responses online. We also post the versions of the paper that were used during peer review. These are the versions that the peer review comments apply to.

The versions of the paper that follow are the versions that were submitted during the peer review process. They are not the versions of record or the final published versions. They should not be cited or distributed as the published version of this manuscript.

BMJ Open is an open access journal and the full, final, typeset and author-corrected version of record of the manuscript is available on our site with no access controls, subscription charges or pay-per-view fees (http://bmjopen.bmj.com).

If you have any questions on BMJ Open's open peer review process please email info.bmjopen@bmj.com

BMJ Open

Protocol for assessment of demographic, epidemiological, and clinical profile of decentralised cancer patients at Nelson Mandela Academic Hospital and Rob Ferreira Hospital, South Africa

Journal:	*BMJ Open*
Manuscript ID	bmjopen-2021-054983
Article Type:	Protocol
Date Submitted by the Author:	01-Jul-2021

Complete List of Authors:	Chitha, Wezile; University of the Witwatersrand Faculty of Health Sciences, Health Systems Enablement and Innovation<br>Mnyaka, Onke; University of the Witwatersrand Faculty of Science, Health Systems Enablement & Innovation<br>Jafta, Zukiswa; University of the Witwatersrand Faculty of Health Sciences, Health Systems Enablement & Innovation<br>Essel, Vivien; University of the Witwatersrand Faculty of Health Sciences, Health Systems Enablement and Innovation<br>Hongoro, Danleen; University of the Witwatersrand Faculty of Health Sciences, Health Systems Enablement and Innovation<br>Godlimpi, Lizo; Walter Sisulu University, Public Health<br>Swartbooi, Buyiswa; University of the Witwatersrand Faculty of Health Sciences, Health Systems Enablement and Innovation<br>Williams, Natasha; University of the Witwatersrand Faculty of Health Sciences, Health Systems Enablement and Innovation<br>Zungu, Christopher; University of the Witwatersrand Faculty of Health Sciences, Health Systems Enablement and Innovation<br>Buthi, Lazola; Nelson Mandela Academic Hospital, Oncology<br>Kuseni, Sibulelo; Nelson Mandela Academic Hospital, Oncology<br>Nasila, John; University of the Witwatersrand Faculty of Health Sciences, Health Systems Enablement and Innovation; Walter Sisulu University, Statistics<br>Sibulawa, Siyabonga; University of the Witwatersrand Faculty of Health Sciences, Health Systems Enablement and Innovation<br>Giwu, Olona; University of the Witwatersrand Faculty of Health Sciences, Health Systems Enablement and Innovation<br>Mavimbela, Awam; University of the Witwatersrand Faculty of Health Sciences, Health Systems Enablement and Innovation<br>Mabunda, Sikhumbuzo; University of New South Wales, The George Institute for Global Health

Keywords:	ONCOLOGY, Cancer pain < ONCOLOGY, CHEMOTHERAPY

For peer review only - http://bmjopen.bmj.com/site/about/guidelines.xhtml

Protocol for assessment of demographic, epidemiological, and clinical profile of decentralised cancer patients at Nelson Mandela Academic Hospital and Rob Ferreira Hospital, South Africa

Wezile Chitha[1], *Onke R. Mnyaka[1], Zukiswa Jafta[1], Vivien Essel[1], Danleen Hongoro[1], Lizo Godlimpi[2], Buyiswa Swartbooi[1], Natasha Williams[1], Christopher Zungu[1], Lazola Buthi[3], Sibulelo Kuseni[3], John Nasila[1,4], Siyabonga Sibulawa[1], Olona Giwu[1], Awam Mavimbela[1], Sikhumbuzo A. Mabunda[1,5]

Author affiliations

[1]Health Systems Enablement & Innovation Unit, University of the Witwatersrand, Johannesburg, South Africa

[2]Department of Public Health, Walter Sisulu University, Mthatha, South Africa

[3]Oncology unit, Nelson Mandela Academic Hospital, Mthatha, South Africa

[4]Department of Statistics, Walter Sisulu University, Mthatha, South Africa

[5] The George Institute for Global Health and Research, University of New South Wales, Sydney, Australia

***Corresponding to:** Onke R. Mnyaka; omnyaka@hsei.co.za; onkemnyaka@gmail.com

Postal address: Queens Office Park, Building 1FF

Alexandra Road, Irene, Centurion, 0157

Tel: +27 11 274 9200

Fax: +27 11 274 9326

Word count: 2739

For peer review only - http://bmjopen.bmj.com/site/about/guidelines.xhtml

ABSTRACT

Introduction

Cancer is the second leading cause of death globally. However, cancer care services are often concentrated in urban centres. Two of South Africa's hospitals have decentralised cancer care delivery from 2019. This study aims to describe the baseline demographic, epidemiological and clinical profile of various cancers at Nelson Mandela Academic hospital (NMAH) and Rob Ferreira hospital (RFH), in South Africa's Eastern Cape and Mpumalanga provinces respectively.

Methods and analysis

This study will be conducted in the Eastern Cape and Mpumalanga provinces. A quantitative, descriptive, exploratory cross-sectional and an ambidirectional cohort study design will be undertaken to gain insight on the baseline characteristics of 424 randomly sampled patients treated for cancer at NMAH and RFH between the 01st of March 2019 and the 28th of February 2022. A validated, researcher-administered survey questionnaire will be used to assess demographic characteristics, and prevalence of different cancers among patients. Concurrently, a document review will be undertaken on cancer patients using the patient registry to ascertain the duration of diagnosis, type of cancer(s), management plan and patient survival time. STATA version 16 will be used for data analysis. The Shapiro-Wilk test will be used to explore the distribution of numerical variables. The Chi-squared or Fisher's exact tests will be used depending on the value of the expected frequencies to compare categorical variables. Kaplan-Meier survival estimates will be used to determine the survival time. Hazard ratios will be used to determine the predictors of death. The level of statistical significance will be set at p-value≤0.05. The 95% confidence interval will be used for the precision of estimates.

Ethics and dissemination

Ethics approval was obtained from the Human Research Ethics Committee of Walter Sisulu University, South Africa (040/2020). Findings will be reported through peer-reviewed journal(s), presentations at conferences and at partner meetings.

Keywords: Cancer, decentralised, referral, oncology; AND South Africa

For peer review only - http://bmjopen.bmj.com/site/about/guidelines.xhtml

Strengths and limitations of this study

- This is the first study to formally report on decentralisation of cancer care services in South Africa.
- Triangulation of designs compensates for the potential limitations of a single design and thus provide more insight on cancer care delivery models in the selected study sites.
- The ambidirectional cohort design does not only enable the assessment of the survival time and predictors of death but also enables the retrospective and prospective follow-up of patients and thus understand their care plans better.
- The study could be limited by the quality of data or poor information systems thus resulting in missing data.

For peer review only

For peer review only - http://bmjopen.bmj.com/site/about/guidelines.xhtml

INTRODUCTION

Cancer is considered to be the number two cause of death globally, accounting for an estimated 9.6 million deaths.[1 2] Africa has the second lowest cancer related deaths contributing 7.1% to the total cancer deaths globally.[3] Of all cancers, breast cancer has the highest incidence (12.5%) followed by lung cancer (12.2%); colorectum cancer (10.7%); prostate cancer (7.8%); stomach cancer (6%); liver cancer (5%); cervical cancer (3.3%); oesophageal cancer (3.3%) with other cancers accounting for 39.1%.[4] Of all the over 9.8 million global cancer related deaths in 2015, lung cancer accounted for the highest mortality rate with 18.2% followed by; colorectum cancer (9.5%); liver cancer (8.4%); stomach cancer (7.8%); breast cancer (6.9%); oesophagus cancer (5.5.5%); pancreatic cancer (4.7%); prostate cancer (3.8%) and other cancers (35.3%).[3] Cancer is expected to continue to rise as part of the epidemiological transition globally, further straining limited healthcare resources.[1] Signs of this prediction have become more visible with rapidly growing global cancer incidence and mortality rates.[4]

A significant proportion of diagnosed cancers are preventable and may be substantially reduced through mitigation of known risk factors.[2] For example, lifestyle related risk factors could be reduced through behaviour changes including the cessation of tobacco use, reducing alcohol consumption, avoiding high fat and low fibre diet, improve physical activity, avoid obesity, and prevent sun exposure.[5] Other risk factors may be reduced through improved awareness of occupational exposure to environmental risk factors.[5] Approximately a third of cancer deaths are due to behavioural and dietary risks, such as, high body mass index, low fruit and vegetable intake, lack of physical activity, tobacco use, and alcohol use.[6] Smoking being the most common preventable cause of premature mortality worldwide contributes to almost 30% of all cancers in high income countries (HICs)[7]. While heavy alcohol drinking is a known cause of liver cancer and it also has links to other cancers such as, cancer of the airway and digestive tracts, breast, and colon[8].

In South Africa cancer is a growing national health and socio-economic concern.[5] According to the International Agency for Research on Cancer (IACR), in 2020, there were 108 168 new cancer cases in South Africa bringing the risk of developing cancer before the age of 75 years to 20.7% (23.6 % male and 18.7% female).[9] In the same year, 56 802 deaths were reported, bringing the risk of dying from cancer before the age of 75 years to 11.8% (13.9% male and 10.4% among female).[9] According to the 2017 national cancer strategic framework of South Africa,[5] the increasing incidence and the mortality rate presents a huge challenge to those

For peer review only - http://bmjopen.bmj.com/site/about/guidelines.xhtml

affected patients and families who are directly affected by the disease especially those who have limited access to care.[5]

In 2016, the top five cancers affecting women in South Africa were, breast cancer (27.1%), cervical cancer (18.7%); colorectal cancer (6.3%); lung cancer (4.9%) and cancer of the uterus (3.9%), while the top five cancers affecting men were, prostate cancer (25.8%); lung cancer (12%); colorectal cancer (7.3%); Kaposi sarcoma (4.9%) and non-Hodgkin's lymphoma (4.1%).[4]

The Nelson Mandela Academic Hospital (NMAH) and Rob Ferreira Hospital (RFH) in the Eastern Cape and Mpumalanga provinces respectively embarked on a decentralised model of cancer care delivery from 01 March 2019. The two hospitals aim to establish centres of excellence in Cancer Care, a network of cancer care satellite sites at district hospital level and community-based cancer care services. The primary goal is to ensure that patients requiring cancer care are able to get their quality care closer to where they live, health professionals are able to screen and diagnose early, unnecessary delays to treatment are reduced and patients get quality palliative care closer to home. Secondary goals include reducing long distance travelled when seeking cancer care, reducing costs of seeking cancer care and reducing unnecessary and/or late referral of cancer patients. However, due to limited resources the current decentralised model of care is only limited to patients requiring chemotherapy. It is hoped that there will be improvement in the patient experience and quality of cancer care and, reduction of morbidity and mortality through the implementation of the decentralised model of cancer care. However, data on patient demographic characteristics and, epidemiological and clinical profile of various cancers is lacking in these two hospitals. This study therefore seeks to conduct a baseline assessment of demographic, epidemiological and clinical profile of various cancers in NMAH and RFH.

Significance

South Africa's cancer services are generally urban-based and located in tertiary and quaternary health centres with an underdeveloped cancer service platform at district hospital and primary care levels. This means that patients needing cancer care have to travel long distances to big cities/towns in order to access basic cancer care. This creates gaps in access and quality of cancer care delivery between urban areas and rural areas. It is therefore envisaged that this study will provide insight on the distribution and types of cancers in areas where there is currently an underestimation of the burden of disease and as a result incorrect understanding

For peer review only - http://bmjopen.bmj.com/site/about/guidelines.xhtml

of the levels of risk within the local populations. Moreover, establish the extent of the problem in both health facilities and possibly justify the need for decentralisation of cancer care services and help inform cancer preventive strategies in South Africa and other similar settings.

Objectives

- To describe the socio-demographic characteristics of patients diagnosed with cancer in selected hospitals in the Eastern Cape and Mpumalanga provinces, South Africa.
- To determine and compare the incidence rate and prevalence of different types of cancer in the selected hospitals.
- To determine and compare the geographic distribution of cancers in the Eastern Cape and Mpumalanga provinces of South Africa.
- To determine the gaps between symptom development, first presentation at a health institution, first cancer diagnosis, referral for definitive management and initiation on treatment of patients diagnosed with cancer in South Africa's Eastern Cape and Mpumalanga provinces.
- To determine the comorbid conditions of patients with a cancer diagnosis in South Africa's Eastern Cape and Mpumalanga provinces.
- To determine the survival time of patients diagnosed with cancer in South Africa's Eastern Cape and Mpumalanga provinces.

METHODS AND ANALYSIS

Research design

This study will utilise a quantitative approach with a triangulation of a descriptive, exploratory cross-sectional and a longitudinal cohort design to answer the study objectives. The triangulation of designs compensates for the potential limitations of a single design. This study forms part of a bigger but yet to be published research project titled: "Exploring the feasibility, implications and outcomes of decentralising cancer care delivery in the Eastern Cape and Mpumalanga provinces of South Africa".

Information will be sourced through two quantitative sub-studies, a cross-sectional survey with cancer patients and an ambi-directional cohort document review. Below is a brief description of the two sub-studies.

For peer review only - http://bmjopen.bmj.com/site/about/guidelines.xhtml

Sub-study 1: Quantitative cross-sectional study

A quantitative survey questionnaire will be administered on patients to assess demographic characteristics, prevalence of different cancers in selected hospitals and, compare geographic distribution of cancers in the Eastern Cape and Mpumalanga provinces.

Sub-study 2: Quantitative ambidirectional cohort study design

Using the cancer patient registry used in the study sites, a document review will be carried out on cancer patients to ascertain the duration of cancer diagnosis, type of cancer(s), and the duration of survival since admission to the oncology clinic (survival time). Figure 1 shows the ambi-directional component of the study.

Table 1 below summarises the two sub-studies.

Table 1: Research methods summary

**Sub-study**	**Study design**	**Objectives**	**Analysis**
1	Cross-sectional study	- Describe socio-demographic characteristics of patients.<br>- Determine and compare the geographic distribution of cancers.<br>- Determine cancer disease progression.<br>- Determine comorbid conditions of cancer patients.	• Frequency tables, percentages, and graphs to summarise categorical variables.<br>• Mean, standard deviation and range to summarise normally distributed numerical variables; or Median and interquartile range to summarise skewed numerical variables.<br>• Chi-squared statistics or Fisher's exact test to compare categorical variables between groups.
2	Ambidirectional cohort study (document review)	- To determine and compare the incidence rate and prevalence of different types of cancer in the selected hospitals.<br>- To determine the comorbid conditions of patients with a cancer diagnosis.	

For peer review only

For peer review only - http://bmjopen.bmj.com/site/about/guidelines.xhtml

		- To determine the survival time of patients diagnosed with cancer.	• Parametric and/or non-parametric tests to compare numerical variables between groups.<br>• Kaplan-Meier survival estimates, for survival time.<br>• Hazard ratios for predictors of death.

Study setting

The study is located in two rural provinces with a high degree of under-development and marginalisation, namely Eastern Cape and Mpumalanga provinces in South Africa.[10] Generally, Eastern Cape and Mpumalanga provinces are characterised by lack of the necessary infrastructure, resources, and expertise to provide quality, safe and accessible radiotherapy, chemotherapy, palliative care services and surgical oncology services. Patients from rural communities, who generally cannot afford private healthcare and are dependent on state health services for cancer care, are compelled to travel long distances to the urban-based tertiary or quaternary cancer care centres in order to access cancer care. The study will be conducted in two hospitals, Nelson Mandela Academic Hospital (NMAH) in Mthatha, Eastern Cape and Rob Ferreira Hospital (RFH) in Mbombela, Mpumalanga respectively. NMAH is one of ten of South Africa's central hospitals and is the only one that is located in a rural area. This level of care is meant to be a quaternary level of care. RFH is a tertiary level of care hospital.

These are two hospitals in their respective provinces that refer their patients to seek quality cancer care in hospitals which are farther away. At times, it takes patients up to 3-days of traveling when attending to their cancer care appointments. For example, cancer patients from Mpumalanga's Rob Ferreira Hospital travel more than 400 kilometres to country's capital city, Pretoria. While cancer patients from the Eastern Cape's Nelson Mandela Academic Hospital travel more than 200 kilometres to East London to access quality cancer care at an urban-based tertiary hospital. An anomaly, as NMAH is statutorily a level of care higher than a tertiary

For peer review only

For peer review only - http://bmjopen.bmj.com/site/about/guidelines.xhtml

hospital. The study hospitals have been selected based on their levels of care, gazetted specialist packages of care and concerns about the existing package of cancer care services.

Population and Sampling

A triangulation of approaches will be used to select study participants from the two hospitals. The study hospitals have been purposely selected because they are currently implementing a decentralised model of cancer care delivery. Furthermore, the two hospitals aim to establish centres of excellence in cancer care, a network of cancer care satellite sites at district hospital level and community-based cancer care services.

Sub-study 1: Quantitative cross-sectional study (patients)

Systematic random sampling of patients visiting the oncology clinics' outpatient's department will be conducted by approaching every 5th patient on the queue until the sampling size has been reached. A total combined sample size for the two hospitals will be calculated using the equation, $n = \frac{p(1-p)z^2}{d^2}$ for a one-sided 95% confidence interval and a 5% significance level (z=1.96). Because the proportion (p) of cancer patients who are seen in the respective hospitals is not known, this (p) will be set at 50% and the margin of error (d) will be set at 5%. This thus yields a total minimum sample size of 385. To factor for data entry errors a further 10% (39) will be added to yield a desired sample size of 424 participants for the two sites. Participants will then be recruited proportionally to yield a sample size of 212 end-user participants per site.

Sub-study 2: Quantitative ambi-directional cohort (document review)

Information will be extracted from the patient registry to respond to the questions on the extraction tool. All patients under the care of the unit at any stage between the 01st of March 2019 and the 28th of February 2022 will be included.

Data collection

A multi-method approach to data collection will be adopted to get a comprehensive picture on cancer in the selected hospitals in terms of demographic distribution of cancer, socio-economic characteristics, prevalence, duration of diagnosis, etc. This approach will also compensate for the potential limitations of a single data collection method and to triangulate the data as a means of checking the consistency of the study findings.

Sub-study 1: Quantitative cross-sectional study (patients)

For peer review only - http://bmjopen.bmj.com/site/about/guidelines.xhtml

The aim of this survey is to assess socioeconomic demographic characteristics of cancer patients, prevalence of different cancers in selected hospitals, compare geographic distribution of the different cancers from the end-user's perspective. This sub-study will adopt and utilise a standardised and validated quantitative survey tool (Appendix A) to collect data from patients. This instrument asks questions on, demographical data and epidemiological and clinical profile of various cancers. This questionnaire will be translated into local languages such as isiXhosa, siSwati, and isiZulu to accommodate participants who might not be comfortable with English.

Sub-study 2: Quantitative ambidirectional cohort study (document review)

Using a data extraction tool (Appendix B), a document review will be conducted in addition to the survey questionnaire. The main aim of the document review is to ascertain information which could not be captured or verified from the survey questionnaire, including, duration of cancer diagnosis, types of cancer, and survival time of cancer patients from the date of diagnosis.

Data management and analysis

Quantitative data analysis will include capturing survey data into Microsoft Excel Office 2016 and exporting the data into STATA version 16.1 (STATA Corp, College Station, Texas, USA) for analysis. Some descriptive and categorical data will be compared using frequencies, percentages, and graphs. Numerical data will be explored for normality using the Shapiro-Wilk test.[11] If normally distributed the mean, range and standard deviation will be used. If not normally distributed, then the median, and interquartile range (IQR) will be used. The Wilcoxon rank sum or an appropriate two-sample t-test will be used to compare the mean or median age of cancer patients by cancer type and between the two sites depending on the normality of the distribution of age and/or the equality of variances. A test for the equality of variances will be performed before use of the two-sample t-tests, if numerical variables are normally distributed. The two-sample t-test for independent samples will be carried out if variances are equal, and Satterthwaite's modified t-test used if the variances are not equal. The Chi-squared or Fisher's exact tests will be used depending on the value of the expected frequencies. If expected frequencies are <5 in binary comparisons or if any one cell of a larger comparison has an expected frequency of <1 or more than 20% of the cells of nominal categorical comparisons have an expected frequency of <5 then the Fisher's exact test will be used. Kaplan-Meier survival estimates will be used to determine the survival time. Hazard ratios will

For peer review only - http://bmjopen.bmj.com/site/about/guidelines.xhtml

be used to determine the predictors of death. The level of statistical significance will be set at p-value ≤ 0.05. The 95% confidence interval will be used for the precision of estimates.

Patient and public involvement

The planning of the cancer service expansion involved community representatives through hospital boards in workshops and meetings. Patients will be informed of the study at all stages through consultations and public notices in the study sites.

Ethics and dissemination

ee-00000 Access approval has been obtained from the Provincial Health Research Committees of the Eastern Cape (EC_202010_012) and Mpumalanga (MP_202011_002) provinces respectively. The study will abide by the 4 ethical principles of autonomy, beneficence, non-maleficence, and justice. Participants will be informed that their participation in this study is voluntary and that their confidentiality will be maintained throughout the study. Participants will also be assured that they are free to withdraw at any stage of the study and could opt-out of questions that they are not comfortable with. All identifying information will be removed. All electronic records will be accessed through a password encrypted database that only the principal investigator has access to. No direct incentives will be issued to participants. Before initiating the self-administered questionnaires, informed consent forms will be signed by all study participants. A waiver of consent has been attained for the document review. Information sheets and consent forms will be translated into relevant local languages (isiXhosa, Siswati and isiZulu). They will also be assured that data collected will be used only for the purposes of the study.

Findings of the study will be disseminated widely to all stakeholders, including participants; and will be used to inform both provincial and national strategies to expand and sustain provision of high-quality cancer screening, diagnosis, treatment, and palliative services, and promote community-based cancer care programmes. Results will be presented at annual partner meetings, national and international conferences. Results will also be published in open access peer-reviewed journals to facilitate broad access to findings.

Authors' contributions

WC conceived the research, sourced funding, engaged stakeholders, completed the first draft of the manuscript and jointly approved final draft. ORM edited and commented on versions of

For peer review only - http://bmjopen.bmj.com/site/about/guidelines.xhtml

the manuscript and incorporated and addressed feedback from the co-authors. SAM edited versions of the manuscript, provided methodological strategy and jointly-approved final draft. ZJ is the content expert, edited and commented on versions of the manuscript. BS facilitated ethics and research access approvals, edited version of the manuscript. VE, DH, LG, NW, CZ, LB, SK, JN, SS, OG and AM edited versions of the manuscript.

Funding statement

This work was supported by the Bristol Myers Squibb Foundation grant number [1028].

Competing interests

None declared

For peer review only

For peer review only - http://bmjopen.bmj.com/site/about/guidelines.xhtml

REFERENCES

1. Fitzmaurice C, Abate D, Abbasi N, et al. Global, Regional, and National Cancer Incidence, Mortality, Years of Life Lost, Years Lived With Disability, and Disability-Adjusted Life-Years for 29 Cancer Groups, 1990 to 2017: A Systematic Analysis for the Global Burden of Disease Study. *JAMA Oncol* 2019;5(12):1749-68. doi: 10.1001/jamaoncol.2019.2996 [published Online First: 2019/09/29]

2. World Health Organisation. Cancer factsheet Geneva: WHO; 2020 [cited 2021 01 June]. Available from: https://www.who.int/news-room/fact-sheets/detail/cancer accessed 01 June 2021.

3. Bray F, Ferlay J, Soerjomataram I, et al. Global cancer statistics 2018: GLOBOCAN estimates of incidence and mortality worldwide for 36 cancers in 185 countries. *CA Cancer J Clin* 2018;68(6):394-424. doi: 10.3322/caac.21492 [published Online First: 2018/09/13]

4. Ferlay J, Colombet M, Soerjomataram I, et al. Estimating the global cancer incidence and mortality in 2018: GLOBOCAN sources and methods. *Int J Cancer* 2019;144(8):1941-53. doi: 10.1002/ijc.31937 [published Online First: 2018/10/24]

5. South African National Departmnet of Health. The National Cancer Strategic Framework 2017 – 2022. Pretoria: NDOH, 2017.

6. Ott JJ, Ullrich A, Mascarenhas M, et al. Global cancer incidence and mortality caused by behavior and infection. *J Public Health (Oxf)* 2011;33(2):223-33. doi: 10.1093/pubmed/fdq076 [published Online First: 2010/10/12]

7. Jha P. The hazards of smoking and the benefits of cessation: a critical summation of the epidemiological evidence in high-income countries. *Elife* 2020;9 doi: 10.7554/eLife.49979 [published Online First: 2020/03/25]

8. Seitz HK, Becker P. Alcohol metabolism and cancer risk. *Alcohol Res Health* 2007;30(1):38-41, 44-7. [published Online First: 2007/08/28]

9. International Agency for Cancer Research. Globocan South Africa Geneva: WHO; 2020 [cited 2021 01 June]. Available from: https://gco.iarc.fr/today/data/factsheets/populations/710-south-africa-fact-sheets.pdf accessed 01 June 2021.

10. Statistics South Africa. Statistics by Place: Census 2011 Pretoria, South Africa: Statistics South Africa; 2020 [Available from: http://www.statssa.gov.za/?page_id=964 accessed 08 June 2021.

11. Vetter TR. Fundamentals of Research Data and Variables: The Devil Is in the Details. *Anesth Analg* 2017;125(4):1375-80. doi: 10.1213/ane.0000000000002370 [published Online First: 2017/08/09]

For peer review only - http://bmjopen.bmj.com/site/about/guidelines.xhtml

Acknowledgements

The authors would like to thank the support received from patient representatives and officials of the participating hospitals and provinces for their assistance.

Patient consent for publication

Not required as this is a protocol. Patients to give consent before enrolment into the study.

Figure 1: Summary of ambi-directional cohort sub-study

For peer review only

For peer review only - http://bmjopen.bmj.com/site/about/guidelines.xhtml

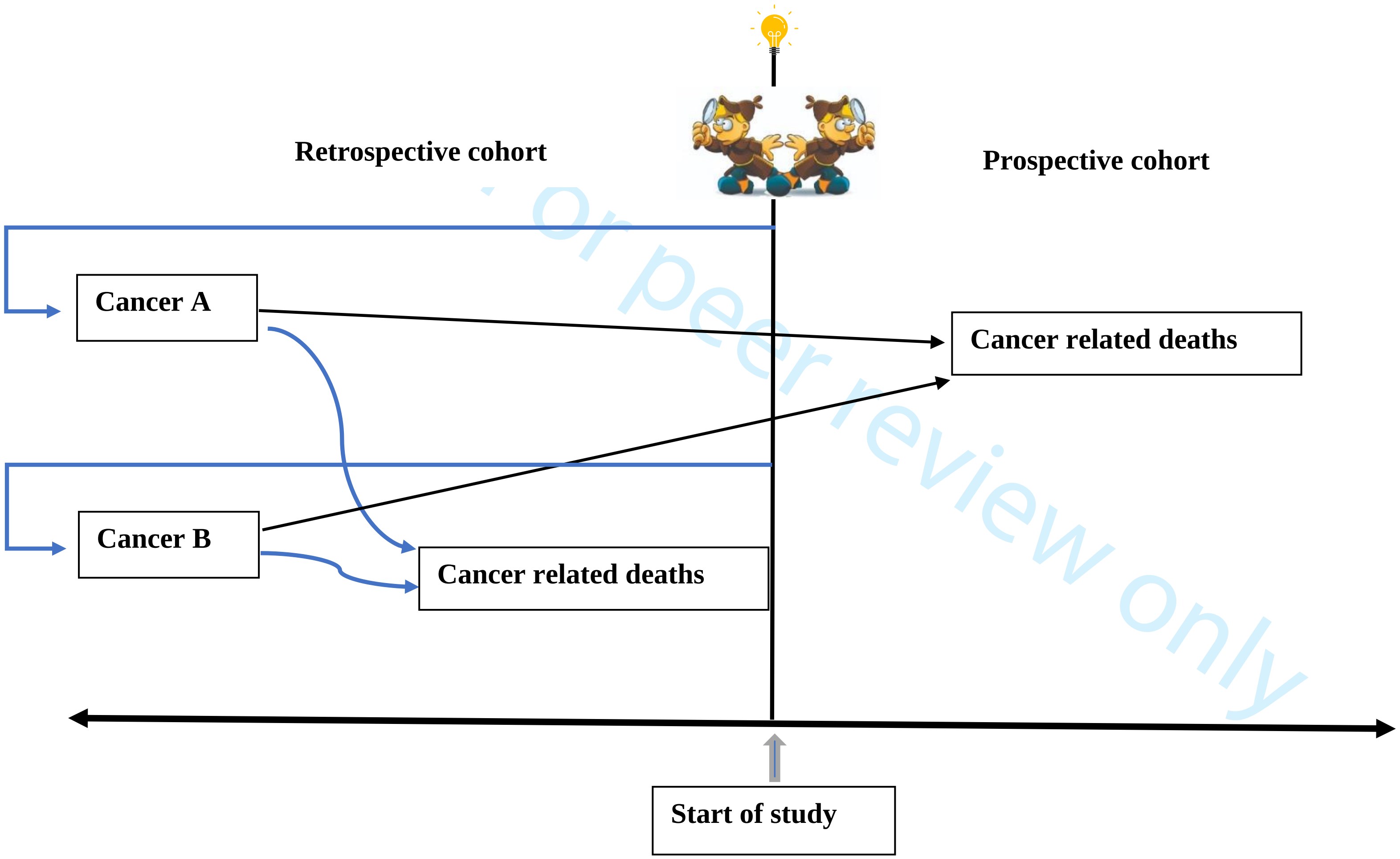

For peer review only - http://bmjopen.bmj.com/site/about/guidelines.xhtml

Appendix A

Survey questionnaire (patients)

INSTRUCTIONS: Fill in the blank spaces with a tick where appropriate.

Date of Administration: ___________________________

Section 1: demographic profile

1. Gender

1	Female	
2	Male	

2. Date of Birth

Dd/Mm/Yy:

3. Ethnicity

1	African	
2	White	
3	Indian	
4	Coloured	
5	Other: specify	

4. Marital status

1	Never Married	
2	Married (including lobola)	
3	Divorced/Separated	
4	Widowed	
5	Cohabiting	

5. What is the highest standard/grade or level you have passed at school or tertiary education?

For peer review only - http://bmjopen.bmj.com/site/about/guidelines.xhtml

6. Are you currently studying?

1	Yes	
2	No	

7. What is your current level of study? --

8. Are you employed?

a)

1	Yes	
2	No	

b) If employed, what type of work do you do? --

9. Source of income (tick all appropriate)

1	2	3	4	5	6	7	8	9
Job	Old Age Grant	Disability Grant	Other Pension	Spousal support	Support from children	Child support grant	None	Other (Specify)

10. What is your gross family income each month (that is, before tax)?

1	No income		7	R6 401 – R12 800	
2	R1 – R400		8	R12 801 – R25 600	
3	R401 – R800		9	R25 601 – R51 200	
4	R801 – R1 600		10	R51 201 – R102 400	
5	R1 601 – R3 200		11	R102 401 – R204 800	
6	R3 201 – R6 400		12	More than R204 800	

11. What is the name of your place of residence?____

12. Which health facility referred you here (Name)?

____

For peer review only - http://bmjopen.bmj.com/site/about/guidelines.xhtml

Section 2: Epidemiological and clinical profile of various cancers

13a. Do you have a family history of cancer?

1	2	3
Yes	No /	Unsure

13b. If yes to above, how are you related to the family member(s) who has the cancer diagnosis?

____
____

13c. What type of cancer(s) does the family member(s) have?

____
____
____

14. Do you smoke? (tick all appropriate)

a)

1	Yes	
2	No	

b) If Yes, when did you start smoking?

Year: __________

c) On average, how many cigarettes do you smoke in a day? ________________

d) If No, have you ever smoked?

1	Yes	
2	No	

e) For how long did you smoke? ___________________

f) How many did you smoke in a day? ________________

1	Once	
2	Twice	
3	Three time	
4	More than 3 times	

For peer review only - http://bmjopen.bmj.com/site/about/guidelines.xhtml

For peer review only

g) Did you stop smoking?

1	Yes	
2	No	

h) How long ago did you stop smoking? ______________________________________

15. Do you drink alcohol?

a)

1	Yes	
2	No	

b) Did you drink alcohol before?

1	Yes	
2	No	

16. Do you exercise (physical) on a regular basis?

1	Yes	
2	No	

17. Have you ever worked in mines?

1	Yes	
2	No	

18. How long in years did you work in mines? --

19. Which mines (tick all that apply)? --

1	Gold	
2	Platinum	
3	diamond	
4	Coal	
5	Other (specify)	

For peer review only - http://bmjopen.bmj.com/site/about/guidelines.xhtml

20. Please indicate if your family has a history of any cancer/s below?

1	Breast cancer		6	Uterus cancer	
2	Lung cancer		7	Colon cancer	
3	Cervical cancer		8	Ovarian cancer	
4	Prostate Cancer		9	Other (specify)	
5	Oesophagus cancer		10	No history of cancer in my family	

21. Before you were told you needed to go to hospital about cancer, how many times did you see other doctors or health professionals about the health problem caused by cancer?

		1x	2x	3x	4x	5x	Other
1	I visited my local clinic						
2	I visited my local hospital						
3	I saw my local private doctor						
4	I saw my traditional healer/doctor/ Isangoma						
5	Other (specify):						

22. How do you feel about the length of time you had to wait before your first appointment with a hospital doctor or clinic doctor?

1	I was seen as soon as I thought was necessary	
2	I should have been seen a bit sooner	

23. How long was it from the time you identified symptoms?

----

24. Did your symptoms get better or worse or were the same while you were waiting for your first appointment with a hospital doctor?

----

25. What type of cancer(s) were you diagnosed with?

----

26. When was your cancer(s) diagnosed?

For peer review only - http://bmjopen.bmj.com/site/about/guidelines.xhtml

27. What health problems or symptoms did you notice at first?

----

28. Who first told you that you had cancer?

1	A hospital doctor	
2	A hospital nurse	
3	A GP (family doctor)	
4	Another health professional (specify)____________	
5	A friend or relative	
6	Nobody – I worked it out for myself	
7	Cannot remember	

29. When you were first told that you had cancer, had you been told you could bring a family member or friend with you?

1	Yes	
2	No	
3	It was not necessary	
4	I was told by phone or letter	
5	Don't know / Can't remember	
6	Other (specify)_________________	

30. How do you feel about the way you were told you had cancer?

----

30. Did you understand the explanation of what was found with you?

1	Yes	
2	No	

31. When you were told you had cancer, were you given written information about the type of cancer you had?

1	Yes	
2	No	

For peer review only - http://bmjopen.bmj.com/site/about/guidelines.xhtml

32. Before your cancer treatment started, were you given a choice of different types of treatment?

1	Yes	
2	No, but I would have liked a choice	
3	I was not given a choice because only one type of treatment was suitable for me	
4	Not sure / Can't remember	
5	Missing	

33. Do you think your views were taken into account when the team of doctors and nurses caring for you were discussing which treatment you should have?

1	Yes	
2	No	

34. Were the possible side effects of treatment(s) explained in a way you could understand?

1	Yes	
2	No	

35. Before you started your treatment, were you given verbal/written information about the side effects of treatment(s)?

1	Yes	
2	No	

36. Were you involved as much as you wanted to be in decisions about your care and treatment?

1	Yes	
2	No	

37a. During the last 12 months, have you had an operation (such as removal of a tumour or lump) at one of the hospitals named in the covering letter?

1	Yes	
2	No	
3	Not sure	

For peer review only - http://bmjopen.bmj.com/site/about/guidelines.xhtml

b. Before you had your operation, did a member of staff explain what would be done during the operation?

1	Yes	
2	No	

38. The last time you went into hospital for a cancer operation, was your admission date changed to a later date by the hospital?

1	Yes	
2	No	

39. Beforehand, were you given written/verbal information about your operation?

1	Yes	
2	No	

40. After the operation, did a member of staff explain how it had gone in a way you could understand?

1	Yes	
2	No	

41. As far as you know, was the hospital or your doctor that referred you for cancer treatment given enough information about your condition and the treatment you had at the hospital?

1	Yes	
2	No	
3	Don’t know / Can’t remember	

42. Do you think the doctors and nurses at your local hospital or clinic did everything they could to support you while you were in their care?

1	Yes	
2	No	

For peer review only - http://bmjopen.bmj.com/site/about/guidelines.xhtml

Appendix B

Document review template

##	Date of birth	Province	Site	Child	Patient Classification	Referred patient	Referral Type	Internal Referral Department	Referring Hospital_ Mpumalanga	- - -
1.										
2.										
3.										
4.										
5.										

##	Referring Hospital_EC	Referral Hospital	Gender	Race	Citizenship	Medical Aid	Postal code	Employed	Source of income	Specify Occupation	- - -
6.											
7.											
8.											
9.											
10.											

##	Previous work in mine	Number of Years worked in mines	Marital Status	Date of 1st oncology visit	Date of diagnosis	Cancer diagnosis 1	ICD10_Cancer diagnosis1	Cancer diagnosis1 _ Stage	Cancer diagnosis 2	ICD10_Cancer diagnosis2	- - -
11.											
12.											
13.											
14.											
15.											

##	Cancer diagnosis2_Stage	Cancer diagnosis3	ICD10_Cancer diagnosis3	Cancer diagnosis3_ Stage	Chemotherapy	Onco_Drug1	Onco_Drug2	Neupogen	Hormonal Therapy	Blood Transfusion	- - -
16.											
17.											
18.											
19.											
20.											

##	Radiotherapy	PET_Bone Scan	Palliative Care	Social support	Psychological support	HIV	Hypertension	Diabetes Mellitus	COPD	Asthma	- - -
21.											
22.											
23.											
24.											
25.											

For peer review only

##	Other Chronic Disease1_Name	Other Chronic Disease2_Name	Family history of cancer	Previous Smoker	Current Smoker	Number of years of smoking	Number of cigarettes smoked in a day	Weight in Kg	Height in Centimetres	Date_of_ Current Visit1	---
26.											
27.											
28.											
29.											
30.											

##	Follow-up Date1	RIP Date	Date_of_Current Visit2	Follow-up Date2	---
31.					
32.					
33.					
34.					
35.					

For peer review only

For peer review only - http://bmjopen.bmj.com/site/about/guidelines.xhtml

BMJ Open

A protocol of mixed-methods assessment of demographic, epidemiological, and clinical profile of decentralised cancer patients at Nelson Mandela Academic Hospital and Rob Ferreira Hospital, South Africa

Journal:	*BMJ Open*
Manuscript ID	bmjopen-2021-054983.R1
Article Type:	Protocol
Date Submitted by the Author:	23-Dec-2021
Complete List of Authors:	Chitha, Wezile; University of the Witwatersrand Faculty of Health Sciences, Health Systems Enablement and Innovation<br>Jafta, Zukiswa; University of the Witwatersrand Faculty of Health Sciences, Health Systems Enablement & Innovation<br>Mnyaka, Onke; University of the Witwatersrand Faculty of Science, Health Systems Enablement & Innovation<br>Hongoro, Danleen; University of the Witwatersrand Faculty of Health Sciences, Health Systems Enablement and Innovation<br>Godlimpi, Lizo; Walter Sisulu University, Public Health<br>Swartbooi, Buyiswa; University of the Witwatersrand Faculty of Health Sciences, Health Systems Enablement and Innovation<br>Williams, Natasha; University of the Witwatersrand Faculty of Health Sciences, Health Systems Enablement and Innovation<br>Zungu, Christopher; University of the Witwatersrand Faculty of Health Sciences, Health Systems Enablement and Innovation<br>Buthi, Lazola; Nelson Mandela Academic Hospital, Oncology<br>Kuseni, Sibulelo; Nelson Mandela Academic Hospital, Oncology<br>Nasila, John; University of the Witwatersrand Faculty of Health Sciences, Health Systems Enablement and Innovation; Walter Sisulu University, Statistics<br>Sibulawa, Siyabonga; University of the Witwatersrand Faculty of Health Sciences, Health Systems Enablement and Innovation<br>Giwu, Olona; University of the Witwatersrand Faculty of Health Sciences, Health Systems Enablement and Innovation<br>Mavimbela, Awam; University of the Witwatersrand Faculty of Health Sciences, Health Systems Enablement and Innovation<br>Essel, Vivien; University of the Witwatersrand Faculty of Health Sciences, Health Systems Enablement and Innovation<br>Mabunda, Sikhumbuzo; University of New South Wales, The George Institute for Global Health; University of New South Wales, School of Population Health
**Primary Subject Heading**:	Oncology
Secondary Subject Heading:	Health services research, Oncology
Keywords:	ONCOLOGY, Cancer pain < ONCOLOGY, CHEMOTHERAPY, Radiation

	oncology < RADIOLOGY & IMAGING

SCHOLARONE™
Manuscripts

A protocol for mixed-methods assessment of demographic, epidemiological, and clinical profile of decentralised cancer patients at Nelson Mandela Academic Hospital and Rob Ferreira Hospital, South Africa

Wezile W. Chitha[1], Zukiswa Jafta[1], *Onke R. Mnyaka[1], Danleen J. Hongoro[1], Lizo Godlimpi[2], Buyiswa Swaartbooi[1], Natasha Williams[1], Christopher Zungu[1], Lazola Buthi[3], Sibulelo Kuseni[3], John Nasila[1,4], Siyabonga Sibulawa[1], Olona Giwu[1], Awam Mavimbela[1], Vivien Essel[1], Sikhumbuzo A. Mabunda[1,5]

Author affiliations

[1]Health Systems Enablement & Innovation Unit, University of the Witwatersrand, Johannesburg, South Africa

[2]Department of Public Health, Walter Sisulu University, Mthatha, South Africa

[3]Oncology unit, Nelson Mandela Academic Hospital, Mthatha, South Africa

[4]Department of Statistics, Walter Sisulu University, Mthatha, South Africa

[5] The George Institute for Global Health and Research, University of New South Wales, Sydney, Australia

[6] School of Population Health, University of New South Wales, Sydney, Australia

¥SAM and VE are joint senior authors

***Corresponding to:** Onke R. Mnyaka; omnyaka@hsei.co.za; onkemnyaka@gmail.com

Postal address: Queens Office Park, Building 1FF

Alexandra Road, Irene, Centurion, 0157

Tel: +27 11 274 9200

Fax: +27 11 274 9326

For peer review only

ABSTRACT

Introduction

Cancer is the second leading cause of death globally. However, cancer care services are often concentrated in urban centres. Two of South Africa's hospitals have decentralised cancer care delivery since February 2018 and August 2019 respectively. This study aims to describe the demographic, epidemiological and clinical profile of various cancers at Nelson Mandela Academic hospital (NMAH) and Rob Ferreira hospital (RFH), in South Africa's Eastern Cape and Mpumalanga provinces respectively.

Methods and analysis

This study will be conducted in the Eastern Cape and Mpumalanga provinces. A mixed methods study design will be undertaken to gain insight on the characteristics of randomly sampled patients that are treated for cancer at NMAH and RFH between the 01st of March 2018 and the 28th of February 2022. A validated, researcher-administered survey questionnaire will be used to assess demographic characteristics, and prevalence of different cancers among patients. Concurrently, a document review will be undertaken on cancer patients using a patient registry to ascertain the duration of diagnosis, type of cancer(s), management plan and patient survival time. STATA version 16 will be used for data analysis. The Shapiro-Wilk test will be used to explore the distribution of numerical variables. The Chi-squared or Fisher's exact tests will be used depending on the value of the expected frequencies to compare categorical variables. Kaplan-Meier survival estimates will be used to determine the survival time. Hazard ratios will be used to determine the predictors of death. The level of statistical significance will be set at p-value ≤0.05. The 95% confidence interval will be used for the precision of estimates.

Ethics and dissemination

Ethics approval was obtained from the Human Research Ethics Committees of the University of the Witwatersrand (M210211) and Walter Sisulu University, South Africa (Ref: 040/2020). Findings will be reported through peer-reviewed journal(s), presentations at conferences and at partner meetings.

For peer review only

Keywords: Cancer, decentralised, referral, oncology; AND South Africa

For peer review only

For peer review only - http://bmjopen.bmj.com/site/about/guidelines.xhtml

Strengths and limitations of this study

- To our knowledge this is the first study to formally report on decentralisation of cancer care services in South Africa.
- Triangulation of designs compensates for the potential limitations of a single design and thus provide more insight on cancer care delivery models in the selected study sites.
- The ambi-directional cohort design does not only enable the assessment of the survival time and predictors of death but also enables the retrospective and prospective follow-up of patients and thus understand their care plans better.
- The study could be limited by the quality of data or poor information systems thus resulting in missing data.

For peer review only

For peer review only - http://bmjopen.bmj.com/site/about/guidelines.xhtml

INTRODUCTION

Cancer is considered to be the number two cause of death globally, accounting for an estimated 9.6 million deaths.[1 2] Africa has the second lowest rate of cancer related deaths contributing 7.1% to the total cancer deaths globally.[3] Cancer is expected to continue to rise as part of the epidemiological transition globally, further straining limited healthcare resources.[1] Signs of this prediction have become more visible with rapidly growing global cancer incidence and mortality rates.[4]

Approximately a third of cancer deaths are due to behavioural and dietary risks, such as, high body mass index, low fruit and vegetable intake, lack of physical activity, tobacco use, and alcohol use.[6] For example, smoking is the most common preventable cause of premature mortality worldwide but it contributes to almost 30% of all cancers in high income countries (HICs).[7] These risk factors are preventable[5] and may be substantially reduced through adjustments in lifestyle.[2]

In South Africa cancer is a growing national health and socio-economic concern.[5] According to the International Agency for Research on Cancer (IACR) in 2020 there were 108 168 new cancer cases in South Africa, bringing the risk of developing cancer before the age of 75 years to 20.7% (23.6 % male and 18.7% female).[9] In the same year, 56 802 deaths were reported, bringing the risk of dying from cancer before the age of 75 years to 11.8% (13.9% male and 10.4% among female).[9] The increasing incidence and mortality rates present a huge challenge to the affected patients and their families especially those who have limited access to care.[5]

In 2016, the top five cancers affecting women in South Africa were, breast cancer (27.1%), cervical cancer (18.7%); colorectal cancer (6.3%); lung cancer (4.9%) and cancer of the uterus (3.9%), while the top five cancers affecting men were, prostate cancer (25.8%); lung cancer (12%); colorectal cancer (7.3%); Kaposi sarcoma (4.9%) and non-Hodgkin's lymphoma (4.1%).[4] The Nelson Mandela Academic Hospital (NMAH), Eastern Cape province and Rob Ferreira Hospital (RFH), Mpumalanga province embarked on a decentralised model of cancer care delivery in February 2018 and August 2019 respectively. Decentralisation refers to making cancer services available in certain hospitals that previously did not have any cancer care service provision, such as our two study sites. In this way, patients can access quality cancer services closest to where they live, health professionals will be able to screen and diagnose early, unnecessary delays to treatment will be reduced and patients will get

For peer review only - http://bmjopen.bmj.com/site/about/guidelines.xhtml

quality palliative care closer to their families. A positive effect of this proposition is that patients' and families' healthcare-related out of pocket costs will be reduced.

The two hospitals aim to establish centres of excellence in cancer care, a network of cancer care satellite sites at district hospital level and community-based cancer care services. Decentralisation is meant to be achieved in four different phases. The first phase entailed decentralisation of chemotherapy services from Frere hospital (East London) and Inkosi Albert Luthuli hospital (Durban, KwaZulu-Natal province) to NMAH in February 2018 for patients from the OR Tambo district (the district where NMAH is located), and three other neighbouring districts for patients in the Eastern Cape province. For Mpumalanga province, all chemotherapy services were decentralised to RFH from Steve Biko and Kalafong hospitals in the Gauteng province in August 2019. The two hospitals (NMAH and RFH) were assisted with the hiring of radiation and medical oncologists, oncology trained professional nurses, pharmacists, social workers, clinical psychologists, ultrasound and mammogram technicians, and administrators. Equipment includes a spirometer, mammogram, ultrasound, colposcopy, and a large loop excision of the transformation zone (LLETZ) machines in both hospitals.

The second phase (current phase) entails decentralisation of chemotherapy services further from NMAH to four Regional hospitals and one District hospital in the Eastern Cape. However, only one of the five hospitals has been fully decentralised from February 2021, the other four hospitals have achieved partial decentralisation with the procurement of equipment. For Mpumalanga, the second phase entails decentralisation of chemotherapy services from RFH to Witbank hospital for patients from two of their three districts from May 2021.

The third phase will be full decentralisation of radiotherapy services from Frere hospital to NMAH in the Eastern Cape and from Steve Biko and Kalafong hospitals to RFH, then Witbank hospital in Mpumalanga. The fourth and final phase is the strengthening of district hospitals and community-based services to manage aspects of cancer effectively from screening, diagnosis, treatment, and palliative care support. This phase will also increase the pool of oncology trained nurses and medical officers at primary care and district hospital level. These latter two phases are still outstanding.

Evidently, the current decentralised model of care is only limited to patients requiring chemotherapy. It is hoped that the continued implementation of the decentralised

For peer review only - http://bmjopen.bmj.com/site/about/guidelines.xhtml

For peer review only

model of cancer care will improve patient experience and quality of cancer care and reduce morbidity and mortality. However, data on patient demographic characteristics, epidemiological and clinical profile of various cancers is lacking in these two hospitals. This study therefore seeks to conduct an assessment of demographic, epidemiological and clinical profile of various cancers in NMAH and RFH. Furthermore, the study aims to describe the current process and its benefits/challenges, with hopes of expanding 'decentralisation of care' in terms of services offered and number of decentralised hospitals.

Significance

South Africa's cancer services are generally urban-based and located in tertiary and quaternary health centres with an underdeveloped cancer service platform at district hospital and primary care levels. This means that patients needing cancer care have to travel long distances to big cities/towns in order to access basic cancer care. This creates gaps in access and quality of cancer care delivery between urban areas and rural areas. Decentralisation is a result of operational observations (to our knowledge there is no formal research that was done) such as long waiting times, delayed presentations, late diagnosis, patient complaints on travelling and out of pocket costs, etc. It is therefore envisaged that this study will provide insight on the distribution and types of cancers in areas where there is currently an underestimation of the burden of disease and as a result incorrect understanding of the levels of risk within the local populations. Moreover, establish the extent of the problem in both health facilities and possibly justify the need for decentralisation of cancer care services and help inform cancer preventive strategies in South Africa and other similar settings.

Objectives

- To describe the socio-demographic characteristics of patients diagnosed with cancer in the selected hospitals in the Eastern Cape and Mpumalanga provinces, South Africa.
- To determine and compare the incidence rate and prevalence of different types of cancer in the selected hospitals.

For peer review only - http://bmjopen.bmj.com/site/about/guidelines.xhtml

- To determine and compare the geographic distribution of cancers in the Eastern Cape and Mpumalanga provinces of South Africa.
- To determine the gaps between symptom development, first presentation at a health institution, first cancer diagnosis, referral for definitive management and initiation on treatment of patients diagnosed with cancer in South Africa's Eastern Cape and Mpumalanga provinces.
- To determine the comorbid conditions of patients with a cancer diagnosis in South Africa's Eastern Cape and Mpumalanga provinces.
- To determine the survival time of patients diagnosed with cancer in South Africa's Eastern Cape and Mpumalanga provinces.

METHODS AND ANALYSIS

Research design

This study will utilise a quantitative approach with a triangulation of a descriptive, exploratory cross-sectional and a longitudinal cohort design to answer the study objectives. The triangulation of designs compensates for the potential limitations of a single design. This study forms part of a bigger but yet to be published research project titled: "Exploring the feasibility, implications and outcomes of decentralising cancer care delivery in the Eastern Cape and Mpumalanga provinces of South Africa".

Information will be sourced through two quantitative sub-studies, a cross-sectional survey with cancer patients and an ambi-directional cohort document review. Below is a brief description of the two sub-studies.

Sub-study 1: Quantitative cross-sectional study

A quantitative survey questionnaire will be administered on patients to assess demographic characteristics, prevalence of different cancers in selected hospitals and compare geographic distribution of cancers in the Eastern Cape and Mpumalanga provinces.

Sub-study 2: Quantitative ambi-directional cohort study design

Using the cancer patient registry used in the study sites, a document review will be carried out on cancer patients to ascertain the duration of cancer diagnosis, type of

For peer review only - http://bmjopen.bmj.com/site/about/guidelines.xhtml

cancer(s), and the duration of survival since admission to the oncology clinic (survival time). Figure 1 shows the ambi-directional component of the study. Table 1 summarises the two sub-studies.

Table 1: Research methods summary

Sub-study	Study design	Objectives	Analysis
1	Cross-sectional study	- Describe socio-demographic characteristics of patients.<br>- Determine and compare the geographic distribution of cancers.<br>- Determine cancer disease progression.<br>- Determine comorbid conditions of cancer patients.	• Frequency tables, percentages, and graphs to summarise categorical variables.<br>• Mean, standard deviation and range to summarise normally distributed numerical variables; or Median and interquartile range to summarise skewed numerical variables.<br>• Chi-squared statistics or Fisher's exact test to compare categorical variables between groups.<br>• Parametric and/or non-parametric tests to compare numerical variables between groups.<br>• Kaplan-Meier survival estimates, for survival time.<br>• Hazard ratios for predictors of death.
2	Ambi-directional cohort study (document review)	- Determine and compare the incidence rate and prevalence of different types of cancer in the selected hospitals.<br>- Determine the comorbid conditions of patients with a cancer diagnosis.<br>- Determine the survival time of patients diagnosed with cancer.	

Study setting

The study is located in two rural provinces with a high degree of under-development and marginalisation, namely Eastern Cape and Mpumalanga provinces in South Africa.[10] Generally, Eastern Cape and Mpumalanga provinces are characterised by

For peer review only - http://bmjopen.bmj.com/site/about/guidelines.xhtml

lack of the necessary infrastructure, resources, and expertise to provide quality, safe and accessible radiotherapy, chemotherapy, palliative care services and surgical oncology services. Patients from rural communities, who generally cannot afford private healthcare and are dependent on state health services for cancer care, are compelled to travel long distances to the urban-based tertiary or quaternary cancer care centres in order to access cancer care. The study will be conducted in two hospitals, NMAH in Mthatha, Eastern Cape province and RFH in Mbombela, Mpumalanga province. NMAH is one of ten central hospitals in South Africa and is the only one that is located in a rural area. This level of care is meant to be a quaternary level of care. RFH is a tertiary level of care hospital.

These are two hospitals in their respective provinces that refer their patients to seek quality cancer care in hospitals which are further away. At times, it takes patients up to 3-days of traveling when attending to their cancer care appointments. For example, cancer patients from Mpumalanga's RFH travel more than 400 kilometres to the country's capital city, Pretoria. While cancer patients from the Eastern Cape's NMAH travel more than 200 kilometres to East London to access quality cancer care at an urban-based tertiary hospital. An anomaly, as NMAH is statutorily a level of care higher than a tertiary hospital.

Population and Sampling

Purposive sampling was used to select the study hospitals. The hospitals were selected based on their levels of care, gazetted specialist packages of care and concerns about the existing package of cancer care services, and because they are currently implementing a decentralised model of cancer care delivery. Furthermore, the two hospitals aim to establish centres of excellence in cancer care, a network of cancer care satellite sites at district hospital level and community-based cancer care services.

A triangulation of approaches will be used to select study participants from the two hospitals.

Sub-study 1: Quantitative cross-sectional study (patients)

For peer review only - http://bmjopen.bmj.com/site/about/guidelines.xhtml

Systematic random sampling of patients visiting the oncology clinics' outpatient's department will be conducted by approaching every 5th patient on the queue until the sampling size has been reached. A total combined sample size for the two hospitals will be calculated using the equation, $n = \frac{p(1-p)z^2}{d^2}$ for a one-sided 95% confidence interval and a 5% significance level (z=1.96). Because the proportion (p) of cancer patients who are seen in the respective hospitals is not known, this (p) will be set at 50% and the margin of error (d) will be set at 5%. This thus yields a total minimum sample size of 385. To factor for data entry errors a further 10% (39) will be added to yield a desired sample size of 424 participants for the two sites. Participants will then be recruited proportionally to yield a sample size of 212 patients per site.

Sub-study 2: Quantitative ambi-directional cohort (document review)

Information will be extracted from the patient registry to respond to the questions on the extraction tool. All patients under the care of the unit at any stage between the 01st of March 2018 and the 28th of February 2022 will be included.

Data collection

A multi-method approach to data collection will be adopted to get a comprehensive picture on cancer in the selected hospitals in terms of demographic distribution of cancer, socio-economic characteristics, prevalence, duration of diagnosis, etc. This approach will also compensate for the potential limitations of a single data collection method and to triangulate the data as a means of checking the consistency of the study findings.

Sub-study 1: Quantitative cross-sectional study (patients)

The aim of this survey is to assess socioeconomic demographic characteristics of cancer patients, prevalence of different cancers in selected hospitals, and compare geographic distribution of the different cancers from the end-user's perspective. This sub-study will adopt and utilise a standardised and validated quantitative survey tool (Appendix A) to collect data from patients. The survey tool for patients has 51 questions developed through literature review and whose content validity was reviewed by three experts (one Occupational medicine specialist, a Public Health Medicine specialist, and an Oncologist). The questionnaire asks about the patient demographic profile and Epidemiological and clinical profile of various cancers. To

For peer review only - http://bmjopen.bmj.com/site/about/guidelines.xhtml

For peer review only

test and ensure the effectiveness, the survey tool was also piloted amongst six patients in the two hospitals. Once the pilot study was done, all necessary adjustments were made to the data collection tool, thus ensuring that all questions will enhance the validity and reliability of the study findings. On clarity, there was 100% agreement among all three experts. On relevance only one of the three experts scored one question as irrelevant to result in an average score Content Validity Index of 0.99 (99%), which still renders the tool valid. Appendix B shows the experts' scoring in detail. This questionnaire has been translated into the local languages (isiXhosa, siSwati, and isiZulu) to accommodate participants who might not be comfortable with English.

Sub-study 2: Quantitative ambi-directional cohort study (document review)

Using a data extraction tool (Appendix C), a document review will be conducted in addition to the survey questionnaire. The main aim of the document review is to ascertain information which could not be captured or verified from the survey questionnaire, including, duration of cancer diagnosis, types of cancer, and survival time of cancer patients from the date of diagnosis.

Data management and analysis

Quantitative data analysis will include capturing survey data into Microsoft Excel Office 2016 and exporting the data into STATA version 17 (STATA Corp, College Station, Texas, USA) for analysis. Some descriptive and categorical data will be compared using frequencies, percentages, and graphs. Numerical data will be explored for normality using the Shapiro-Wilk test.[11] If normally distributed the mean, range and standard deviation will be used. If not normally distributed, then the median, and interquartile range (IQR) will be used. The Wilcoxon rank sum or an appropriate two-sample t-test will be used to compare the mean or median age of cancer patients by cancer type and between the two sites depending on the normality of the distribution of age and/or the equality of variances. A test for the equality of variances will be performed before use of the two-sample t-tests, if numerical variables are normally distributed. The two-sample t-test for independent samples will be carried out if variances are equal, and Satterthwaite's modified t-test used if the variances are not equal. The Chi-squared or Fisher's exact tests will be used depending on the value of the expected frequencies. If expected frequencies are <5 in binary comparisons or if

For peer review only - http://bmjopen.bmj.com/site/about/guidelines.xhtml

any one cell of a larger comparison has an expected frequency of <1 or more than 20% of the cells of nominal categorical comparisons have an expected frequency of <5 then the Fisher's exact test will be used. Kaplan-Meier survival estimates will be used to determine the survival time. Hazard ratios will be used to determine the predictors of death. The level of statistical significance will be set at p-value ≤0.05. The 95% confidence interval will be used for the precision of estimates. Through triangulation, the analysis of the survey will seek to reproduce the cancer prevalence, epidemiological profiles and demographic characteristics determined in sub-study 1. These should be similar as determined by statistical methods (95% confidence interval and two-sample test of proportions).

Limitations

The study could be limited by the quality of data or poor information systems thus resulting in missing data. The assessment of the data at three time points will allow for the reduction of missing data as we will note suspicious entries and/or missing data and request for assistance with correction/filling of the missing data using the patient records or confirming from patients and/or family members. Missing data will be analysed using complete case analysis. The main outcome (cancer related deaths) could be limited by the absence of a mechanism linking to the death certificate, and/or autopsy. Every unspecified natural cause of death will therefore be considered to be related to the cancer as a direct or associated cause of death.

Patient and public involvement

The planning of the cancer service expansion involved community representatives through hospital boards in workshops and meetings. Patients will be informed of the study at all stages through consultations and public notices in the study sites.

Ethics and dissemination

Ethics approval was obtained from the Human Research Ethics Committees of the University of the Witwatersrand (M210211) and Walter Sisulu University, South Africa (040/2020). Site access approval has been obtained from the Provincial Health Research Committees of the Eastern Cape (EC_202010_012) and Mpumalanga (MP_202011_002) provinces respectively. The study will abide by the 4 ethical

For peer review only - http://bmjopen.bmj.com/site/about/guidelines.xhtml

principles of autonomy, beneficence, non-maleficence, and justice. Participants will be informed that their participation in this study is voluntary and that their confidentiality will be maintained throughout the study. Participants will also be assured that they are free to withdraw at any stage of the study and could opt-out of questions that they are not comfortable with. All identifying information will be removed. All electronic records will be accessed through a password encrypted database that only the principal investigator has access to. No direct incentives will be issued to participants. Before initiating the self-administered questionnaires, informed consent forms will be signed by all study participants. A waiver of consent has been attained for the document review. Information sheets and consent forms will be translated into relevant local languages (isiXhosa, Siswati and isiZulu). They will also be assured that data collected will be used only for the purposes of the study.

Findings of the study will be disseminated widely to all stakeholders, including participants; and will be used to inform both provincial and national strategies to expand and sustain provision of high-quality cancer screening, diagnosis, treatment, and palliative services, and promote community-based cancer care programmes. Results will be presented at annual partner meetings, national and international conferences. Results will also be published in open access peer-reviewed journals to facilitate broad access to findings.

Authors' contributions

WC conceived the research, sourced funding, engaged stakeholders, completed the first draft of the manuscript and jointly approved final draft. ORM edited and commented on versions of the manuscript and incorporated and addressed feedback from the co-authors. SAM edited versions of the manuscript, provided methodological strategy, validated the quantitative survey tool, and jointly approved final draft. ZJ is the content expert, edited and commented on versions of the manuscript. BS facilitated ethics and research access approvals, edited version of the manuscript. VE validated the quantitative survey tool and edited versions of the manuscript. DH, LG, NW, CZ, LB, SK, JN, SS, OG and AM edited versions of the manuscript.

For peer review only - http://bmjopen.bmj.com/site/about/guidelines.xhtml

Funding statement

This work was supported by the Bristol Myers Squibb Foundation grant number [1028].

Competing interests

None declared

For peer review only

REFERENCES

1. Fitzmaurice C, Abate D, Abbasi N, et al. Global, Regional, and National Cancer Incidence, Mortality, Years of Life Lost, Years Lived With Disability, and Disability-Adjusted Life-Years for 29 Cancer Groups, 1990 to 2017: A Systematic Analysis for the Global Burden of Disease Study. *JAMA Oncol* 2019;5(12):1749-68. doi: 10.1001/jamaoncol.2019.2996 [published Online First: 2019/09/29]

2. World Health Organisation. Cancer factsheet Geneva: WHO; 2020 [cited 2021 01 June]. Available from: https://www.who.int/news-room/fact-sheets/detail/cancer accessed 01 June 2021.

3. Bray F, Ferlay J, Soerjomataram I, et al. Global cancer statistics 2018: GLOBOCAN estimates of incidence and mortality worldwide for 36 cancers in 185 countries. *CA Cancer J Clin* 2018;68(6):394-424. doi: 10.3322/caac.21492 [published Online First: 2018/09/13]

4. Ferlay J, Colombet M, Soerjomataram I, et al. Estimating the global cancer incidence and mortality in 2018: GLOBOCAN sources and methods. *Int J Cancer* 2019;144(8):1941-53. doi: 10.1002/ijc.31937 [published Online First: 2018/10/24]

5. South African National Departmnet of Health. The National Cancer Strategic Framework 2017 – 2022. Pretoria: NDOH, 2017.

6. Ott JJ, Ullrich A, Mascarenhas M, et al. Global cancer incidence and mortality caused by behavior and infection. *J Public Health (Oxf)* 2011;33(2):223-33. doi: 10.1093/pubmed/fdq076 [published Online First: 2010/10/12]

7. Jha P. The hazards of smoking and the benefits of cessation: a critical summation of the epidemiological evidence in high-income countries. *Elife* 2020;9 doi: 10.7554/eLife.49979 [published Online First: 2020/03/25]

8. Seitz HK, Becker P. Alcohol metabolism and cancer risk. Alcohol Res Health 2007;30(1):38-41, 44-7. [published Online First: 2007/08/28]

9. International Agency for Cancer Research. Globocan South Africa Geneva: WHO; 2020 [cited 2021 01 June]. Available from: https://gco.iarc.fr/today/data/factsheets/populations/710-south-africa-fact-sheets.pdf accessed 01 June 2021.

10. Statistics South Africa. Statistics by Place: Census 2011 Pretoria, South Africa: Statistics South Africa; 2020 [Available from: http://www.statssa.gov.za/?page_id=964 accessed 08 June 2021.

11. Vetter TR. Fundamentals of Research Data and Variables: The Devil Is in the Details. *Anesth Analg* 2017;125(4):1375-80. doi: 10.1213/ane.0000000000002370 [published Online First: 2017/08/09]

For peer review only - http://bmjopen.bmj.com/site/about/guidelines.xhtml

Acknowledgements

The authors would like to thank the support received from patient representatives and officials of the participating hospitals and provinces for their assistance.

Patient consent for publication

Not required as this is a protocol. Patients to give consent before enrolment into the study.

Figure 1: Summary of ambi-directional cohort sub-study

Figure 1: Ambi-directional cohort study for sub-study 2.

Note. Data collection will commence in January 2022 till the end of September 2022. Record review of patients diagnosed with cancer from 1 March 2018 will be done. Patients will be followed up for survival time either retrospectively (cancer related deaths between 1 March 2018 to January 2022), or prospectively (cancer related deaths from January 2022 until the study's end date, 30 September 2022).

For peer review only

For peer review only - http://bmjopen.bmj.com/site/about/guidelines.xhtml

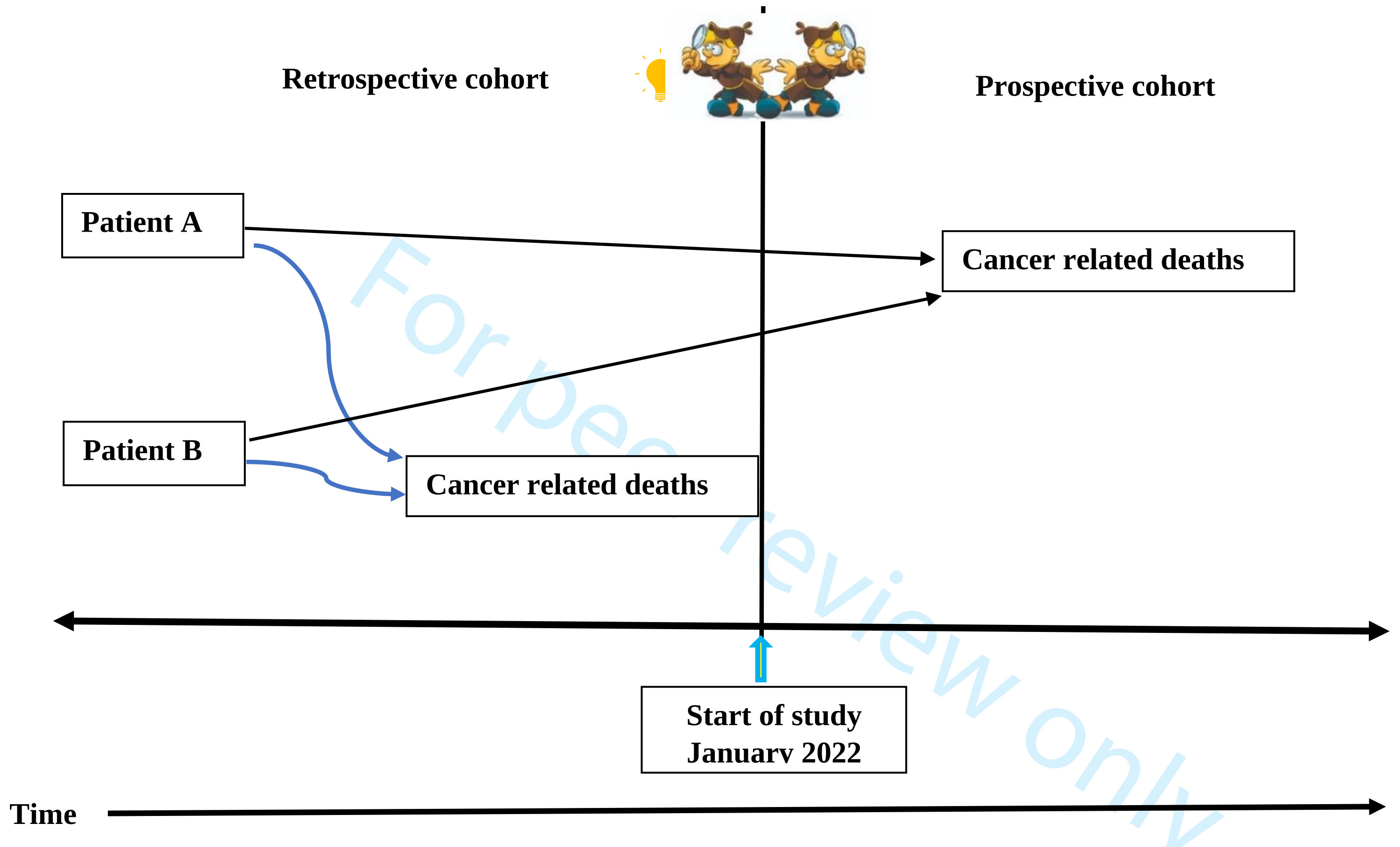

Figure 1: Ambi-directional cohort study for sub-study 2.

Note. Data collection will commence in January 2022 till the end of September 2022. Record review of patients diagnosed with cancer from 1 March 2018 will be done. Patients will be followed up for survival time either retrospectively (cancer related deaths between 1 March 2018 to January 2022), or prospectively (cancer related deaths from January 2022 until the study's end date, 30 September 2022).

Appendix A

Survey questionnaire (patients)

INSTRUCTIONS: Fill in the blank spaces with a tick where appropriate.

Date of Administration: ___________________________

Section 1: demographic profile

1. Gender

1	Female	
2	Male	

2. Date of Birth

Dd/Mm/Yy: | | | | | | | | |

3. Ethnicity

1	African	
2	White	
3	Indian	
4	Coloured	
5	Other: specify	

4. Marital status

1	Never Married	
2	Married (including lobola)	
3	Divorced/Separated	
4	Widowed	
5	Cohabiting	

5. What is the highest standard/grade or level you have passed at school or tertiary education?

For peer review only - http://bmjopen.bmj.com/site/about/guidelines.xhtml

6. Are you currently studying?

1	Yes	
2	No	

7. What is your current level of study? ---

8. Are you employed?

a)

1	Yes	
2	No	

b) If employed, what type of work do you do? --

9. Source of income (tick all appropriate)

1	2	3	4	5	6	7	8	9
Job	Old Age Grant	Disability Grant	Other Pension	Spousal support	Support from children	Child support grant	None	Other (Specify)

10. What is your gross family income each month (that is, before tax)?

1	No income		7	R6 401 – R12 800	
2	R1 – R400		8	R12 801 – R25 600	
3	R401 – R800		9	R25 601 – R51 200	
4	R801 – R1 600		10	R51 201 – R102 400	
5	R1 601 – R3 200		11	R102 401 – R204 800	
6	R3 201 – R6 400		12	More than R204 800	

11. What is the name of your place of residence?____

12. Which health facility referred you here (Name)?

____

For peer review only - http://bmjopen.bmj.com/site/about/guidelines.xhtml

Section 2: Epidemiological and clinical profile of various cancers

13a. Do you have a family history of cancer?

1	2	3
Yes	No /	Unsure

13b. If yes to above, how are you related to the family member(s) who has the cancer diagnosis?

____

____

13c. What type of cancer(s) does the family member(s) have?

____

____

____

14. Do you smoke? (tick all appropriate)

a)

1	Yes	
2	No	

b) If Yes, when did you start smoking?

Year: __________

c) On average, how many cigarettes do you smoke in a day? ____________________

d) If No, have you ever smoked?

1	Yes	
2	No	

e) For how long did you smoke? __________________

f) How many did you smoke in a day? ________________

1	Once	
2	Twice	
3	Three time	
4	More than 3 times	

For peer review only - http://bmjopen.bmj.com/site/about/guidelines.xhtml

For peer review only

g) Did you stop smoking?

1	Yes	
2	No	

h) How long ago did you stop smoking? ____

15. Do you drink alcohol?

a)

1	Yes	
2	No	

b) Did you drink alcohol before?

1	Yes	
2	No	

16. Do you exercise (physical) on a regular basis?

1	Yes	
2	No	

17. Have you ever worked in mines?

1	Yes	
2	No	

18. How long in years did you work in mines? --

19. Which mines (tick all that apply)? --

1	Gold	
2	Platinum	
3	diamond	
4	Coal	
5	Other (specify)	

For peer review only - http://bmjopen.bmj.com/site/about/guidelines.xhtml

For peer review only

20. Please indicate if your family has a history of any cancer/s below?

1	Breast cancer		6	Uterus cancer	
2	Lung cancer		7	Colon cancer	
3	Cervical cancer		8	Ovarian cancer	
4	Prostate Cancer		9	Other (specify)	
5	Oesophagus cancer		10	No history of cancer in my family	

21. Before you were told you needed to go to hospital about cancer, how many times did you see other doctors or health professionals about the health problem caused by cancer?

		1x	2x	3x	4x	5x	Other
1	I visited my local clinic						
2	I visited my local hospital						
3	I saw my local private doctor						
4	I saw my traditional healer/doctor/ Isangoma						
5	Other (specify):						

22. How do you feel about the length of time you had to wait before your first appointment with a hospital doctor or clinic doctor?

1	I was seen as soon as I thought was necessary	
2	I should have been seen a bit sooner	

23. How long was it from the time you identified symptoms?

----

24. Did your symptoms get better or worse or were the same while you were waiting for your first appointment with a hospital doctor?

----

25. What type of cancer(s) were you diagnosed with?

----

26. When was your cancer(s) diagnosed?

For peer review only - http://bmjopen.bmj.com/site/about/guidelines.xhtml

27. What health problems or symptoms did you notice at first?

----

28. Who first told you that you had cancer?

1	A hospital doctor	
2	A hospital nurse	
3	A GP (family doctor)	
4	Another health professional (specify)____________	
5	A friend or relative	
6	Nobody – I worked it out for myself	
7	Cannot remember	

29. When you were first told that you had cancer, had you been told you could bring a family member or friend with you?

1	Yes	
2	No	
3	It was not necessary	
4	I was told by phone or letter	
5	Don't know / Can't remember	
6	Other (specify)_________________	

30. How do you feel about the way you were told you had cancer?

----

30. Did you understand the explanation of what was found with you?

1	Yes	
2	No	

31. When you were told you had cancer, were you given written information about the type of cancer you had?

1	Yes	
2	No	

For peer review only - http://bmjopen.bmj.com/site/about/guidelines.xhtml

For peer review only

32. Before your cancer treatment started, were you given a choice of different types of treatment?

1	Yes	
2	No, but I would have liked a choice	
3	I was not given a choice because only one type of treatment was suitable for me	
4	Not sure / Can't remember	
5	Missing	

33. Do you think your views were taken into account when the team of doctors and nurses caring for you were discussing which treatment you should have?

1	Yes	
2	No	

34. Were the possible side effects of treatment(s) explained in a way you could understand?

1	Yes	
2	No	

35. Before you started your treatment, were you given verbal/written information about the side effects of treatment(s)?

1	Yes	
2	No	

36. Were you involved as much as you wanted to be in decisions about your care and treatment?

1	Yes	
2	No	

37a. During the last 12 months, have you had an operation (such as removal of a tumour or lump) at one of the hospitals named in the covering letter?

1	Yes	
2	No	
3	Not sure	

For peer review only - http://bmjopen.bmj.com/site/about/guidelines.xhtml

b. Before you had your operation, did a member of staff explain what would be done during the operation?

1	Yes	
2	No	

38. The last time you went into hospital for a cancer operation, was your admission date changed to a later date by the hospital?

1	Yes	
2	No	

39. Beforehand, were you given written/verbal information about your operation?

1	Yes	
2	No	

40. After the operation, did a member of staff explain how it had gone in a way you could understand?

1	Yes	
2	No	

41. As far as you know, was the hospital or your doctor that referred you for cancer treatment given enough information about your condition and the treatment you had at the hospital?

1	Yes	
2	No	
3	Don't know / Can't remember	

42. Do you think the doctors and nurses at your local hospital or clinic did everything they could to support you while you were in their care?

1	Yes	
2	No	

For peer review only

For peer review only - http://bmjopen.bmj.com/site/about/guidelines.xhtml

Appendix B

Document review template

##	Date of birth	Province	Site	Child	Patient Classification	Referred patient	Referral Type	Internal Referral Department	Referring Hospital_ Mpumalanga	---
1.										
2.										
3.										
4.										
5.										

##	Referring Hospital_EC	Referral Hospital	Gender	Race	Citizenship	Medical Aid	Postal code	Employed	Source of income	Specify Occupation	---
6.											
7.											
8.											
9.											
10.											

##	Previous work in mine	Number of Years worked in mines	Marital Status	Date of 1st oncology visit	Date of diagnosis	Cancer diagnosis 1	ICD10_Cancer diagnosis1	Cancer diagnosis1_ Stage	Cancer diagnosis 2	ICD10_Cancer diagnosis2	---
11.											
12.											
13.											
14.											
15.											

##	Cancer diagnosis2_Stage	Cancer diagnosis3	ICD10_Cancer diagnosis3	Cancer diagnosis3_ Stage	Chemotherapy	Onco_Drug1	Onco_Drug2	Neupogen	Hormonal Therapy	Blood Transfusion	---
16.											
17.											
18.											
19.											
20.											

##	Radiotherapy	PET_Bone Scan	Palliative Care	Social support	Psychological support	HIV	Hypertension	Diabetes Mellitus	COPD	Asthma	---
21.											
22.											
23.											
24.											
25.											

For peer review only - http://bmjopen.bmj.com/site/about/guidelines.xhtml

##	Other Chronic Disease1_Name	Other Chronic Disease2_Name	Family history of cancer	Previous Smoker	Current Smoker	Number of years of smoking	Number of cigarettes smoked in a day	Weight in Kg	Height in Centimetres	Date_of_ Current Visit1	---
26.											
27.											
28.											
29.											
30.											

##	Follow-up Date1	RIP Date	Date_of_Current Visit2	Follow-up Date2	---
31.					
32.					
33.					
34.					
35.					

For peer review only

For peer review only - http://bmjopen.bmj.com/site/about/guidelines.xhtml

BMJ Open

A protocol of mixed-methods assessment of demographic, epidemiological, and clinical profile of decentralised cancer patients at Nelson Mandela Academic Hospital and Rob Ferreira Hospital, South Africa

Journal:	*BMJ Open*
Manuscript ID	bmjopen-2021-054983.R2
Article Type:	Protocol
Date Submitted by the Author:	22-Feb-2022
Complete List of Authors:	Chitha, Wezile; University of the Witwatersrand Faculty of Health Sciences, Health Systems Enablement and Innovation<br>Jafta, Zukiswa; University of the Witwatersrand Faculty of Health Sciences, Health Systems Enablement & Innovation<br>Mnyaka, Onke; University of the Witwatersrand Faculty of Science, Health Systems Enablement & Innovation<br>Hongoro, Danleen; University of the Witwatersrand Faculty of Health Sciences, Health Systems Enablement and Innovation<br>Godlimpi, Lizo; Walter Sisulu University, Public Health<br>Swartbooi, Buyiswa; University of the Witwatersrand Faculty of Health Sciences, Health Systems Enablement and Innovation<br>Williams, Natasha; University of the Witwatersrand Faculty of Health Sciences, Health Systems Enablement and Innovation<br>Zungu, Christopher; University of the Witwatersrand Faculty of Health Sciences, Health Systems Enablement and Innovation<br>Buthi, Lazola; Nelson Mandela Academic Hospital, Oncology<br>Kuseni, Sibulelo; Nelson Mandela Academic Hospital, Oncology<br>Nasila, John; University of the Witwatersrand Faculty of Health Sciences, Health Systems Enablement and Innovation; Walter Sisulu University, Statistics<br>Sibulawa, Siyabonga; University of the Witwatersrand Faculty of Health Sciences, Health Systems Enablement and Innovation<br>Giwu, Olona; University of the Witwatersrand Faculty of Health Sciences, Health Systems Enablement and Innovation<br>Mavimbela, Awam; University of the Witwatersrand Faculty of Health Sciences, Health Systems Enablement and Innovation<br>Essel, Vivien; University of the Witwatersrand Faculty of Health Sciences, Health Systems Enablement and Innovation<br>Mabunda, Sikhumbuzo; University of New South Wales, The George Institute for Global Health; University of New South Wales, School of Population Health
**Primary Subject Heading**:	Oncology
Secondary Subject Heading:	Health services research, Oncology
Keywords:	ONCOLOGY, Cancer pain < ONCOLOGY, CHEMOTHERAPY, Radiation

	oncology < RADIOLOGY & IMAGING

SCHOLARONE™ Manuscripts

A protocol of mixed-methods assessment of demographic, epidemiological, and clinical profile of decentralised cancer patients at Nelson Mandela Academic Hospital and Rob Ferreira Hospital, South Africa

Wezile W. Chitha[1], Zukiswa Jafta[1], *Onke R. Mnyaka[1], Danleen J. Hongoro[1], Lizo Godlimpi[2], Buyiswa Swaartbooi[1], Natasha Williams[1], Christopher Zungu[1], Lazola Buthi[3], Sibulelo Kuseni[3], John Nasila[1,4], Siyabonga Sibulawa[1], Olona Giwu[1], Awam Mavimbela[1], Vivien Essel[1], Sikhumbuzo A. Mabunda[1,5, 6]

Author affiliations

[1]Health Systems Enablement & Innovation Unit, University of the Witwatersrand, Johannesburg, South Africa

[2]Department of Public Health, Walter Sisulu University, Mthatha, South Africa

[3]Oncology unit, Nelson Mandela Academic Hospital, Mthatha, South Africa

[4]Department of Statistics, Walter Sisulu University, Mthatha, South Africa

[5] The George Institute for Global Health and Research, University of New South Wales, Sydney, Australia

[6] School of Population Health, University of New South Wales, Sydney, Australia

¥SAM and VE are joint senior authors

***Corresponding to:** Onke R. Mnyaka; omnyaka@hsei.co.za; onkemnyaka@gmail.com

Postal address: Queens Office Park, Building 1FF

Alexandra Road, Irene, Centurion, 0157

Tel: +27 11 274 9200

Fax: +27 11 274 9326

For peer review only

For peer review only - http://bmjopen.bmj.com/site/about/guidelines.xhtml

ABSTRACT

Introduction

Cancer is the second leading cause of death globally. However, cancer care services are often concentrated in urban centres. Two of South Africa's hospitals have decentralised cancer care delivery since February 2018 and August 2019 respectively. This study aims to describe the demographic, epidemiological and clinical profile of various cancers at Nelson Mandela Academic hospital (NMAH) and Rob Ferreira hospital (RFH), in South Africa's Eastern Cape and Mpumalanga provinces respectively.

Methods and analysis

This study will be conducted in the Eastern Cape and Mpumalanga provinces. A mixed methods study design will be undertaken to gain insight on the characteristics of randomly sampled patients that are treated for cancer at NMAH and RFH between the 01st of March 2018 and the 28th of February 2022. A validated, researcher-administered survey questionnaire will be used to assess demographic characteristics, and prevalence of different cancers among patients. Concurrently, a document review will be undertaken on cancer patients using a patient registry to ascertain the duration of diagnosis, type of cancer(s), management plan and patient survival time. STATA version 16 will be used for data analysis. The Shapiro-Wilk test will be used to explore the distribution of numerical variables. The Chi-squared or Fisher's exact tests will be used depending on the value of the expected frequencies to compare categorical variables. Kaplan-Meier survival estimates will be used to determine the survival time. Hazard ratios will be used to determine the predictors of death. The level of statistical significance will be set at p-value ≤ 0.05. The 95% confidence interval will be used for the precision of estimates.

Ethics and dissemination

Ethics approval was obtained from the Human Research Ethics Committees of the University of the Witwatersrand (M210211) and Walter Sisulu University, South Africa (Ref: 040/2020). Findings will be reported through peer-reviewed journal(s), presentations at conferences and at partner meetings.

Keywords: Cancer, decentralised, referral, oncology; AND South Africa

For peer review only - http://bmjopen.bmj.com/site/about/guidelines.xhtml

Strengths and limitations of this study

- To our knowledge this is the first study to formally report on decentralisation of cancer care services in South Africa.
- Triangulation of designs compensates for the potential limitations of a single design and thus provide more insight on cancer care delivery models in the selected study sites.
- The ambi-directional cohort design does not only enable the assessment of the survival time and predictors of death but also enables the retrospective and prospective follow-up of patients and thus understand their care plans better.
- The study could be limited by the quality of data or poor information systems thus resulting in missing data.

For peer review only

For peer review only - http://bmjopen.bmj.com/site/about/guidelines.xhtml

INTRODUCTION

Cancer is considered to be the number two cause of death globally, accounting for an estimated 9.6 million deaths (1)(2). Africa has the second lowest rate of cancer related deaths contributing 7.1% to the total cancer deaths globally (3). Cancer is expected to continue to rise as part of the epidemiological transition globally, further straining limited healthcare resources (1). Signs of this prediction have become more visible with rapidly growing global cancer incidence and mortality rates (3).

Approximately a third of cancer deaths are due to behavioural and dietary risks, such as, high body mass index, low fruit and vegetable intake, lack of physical activity, tobacco use, and alcohol use (4). For example, smoking is the most common preventable cause of premature mortality worldwide but it contributes to almost 30% of all cancers in high income countries (HICs) (5). These risk factors are preventable (6) and may be substantially reduced through adjustments in lifestyle (2).

In South Africa cancer is a growing national health and socio-economic concern (6). According to the International Agency for Research on Cancer (IACR) in 2020 there were 108 168 new cancer cases in South Africa, bringing the risk of developing cancer before the age of 75 years to 20.7% (23.6 % male and 18.7% female) (7). In the same year, 56 802 deaths were reported, bringing the risk of dying from cancer before the age of 75 years to 11.8% (13.9% male and 10.4% among female) (7). The increasing incidence and mortality rates present a huge challenge to the affected patients and their families especially those who have limited access to care (6).

In 2016, the top five cancers affecting women in South Africa were, breast cancer (27.1%), cervical cancer (18.7%); colorectal cancer (6.3%); lung cancer (4.9%) and cancer of the uterus (3.9%), while the top five cancers affecting men were, prostate cancer (25.8%); lung cancer (12%); colorectal cancer (7.3%); Kaposi sarcoma (4.9%) and non-Hodgkin's lymphoma (4.1%) (3). The Nelson Mandela Academic Hospital (NMAH), Eastern Cape province and Rob Ferreira Hospital (RFH), Mpumalanga province embarked on a decentralised model of cancer care delivery in February 2018 and August 2019 respectively. Decentralisation refers to making cancer services available in certain hospitals that previously did not have any cancer care service provision, such as our two study sites. In this way, patients can access quality cancer services closest to where they live, health professionals will be able to screen and diagnose early, unnecessary delays to treatment will be reduced and patients will get quality palliative care closer to their families. A positive effect of this proposition is that patients' and families' healthcare-related out of pocket costs will be reduced.

For peer review only - http://bmjopen.bmj.com/site/about/guidelines.xhtml

The two hospitals aim to establish centres of excellence in cancer care, a network of cancer care satellite sites at district hospital level and community-based cancer care services. Decentralisation is meant to be achieved in four different phases. The first phase entailed decentralisation of chemotherapy services from Frere hospital (East London) and Inkosi Albert Luthuli hospital (Durban, KwaZulu-Natal province) to NMAH in February 2018 for patients from the OR Tambo district (the district where NMAH is located), and three other neighbouring districts for patients in the Eastern Cape province. For Mpumalanga province, all chemotherapy services were decentralised to RFH from Steve Biko and Kalafong hospitals in the Gauteng province in August 2019. The two hospitals (NMAH and RFH) were assisted with the hiring of radiation and medical oncologists, oncology trained professional nurses, pharmacists, social workers, clinical psychologists, ultrasound and mammogram technicians, and administrators. Equipment includes a spirometer, mammogram, ultrasound, colposcopy, and a large loop excision of the transformation zone (LLETZ) machines in both hospitals.

The second phase (current phase) entails decentralisation of chemotherapy services further from NMAH to four Regional hospitals and one District hospital in the Eastern Cape. However, only one of the five hospitals has been fully decentralised from February 2021, the other four hospitals have achieved partial decentralisation with the procurement of equipment. For Mpumalanga, the second phase entails decentralisation of chemotherapy services from RFH to Witbank hospital for patients from two of their three districts from May 2021.

The third phase will be full decentralisation of radiotherapy services from Frere hospital to NMAH in the Eastern Cape and from Steve Biko and Kalafong hospitals to RFH, then Witbank hospital in Mpumalanga. The fourth and final phase is the strengthening of district hospitals and community-based services to manage aspects of cancer effectively from screening, diagnosis, treatment, and palliative care support. This phase will also increase the pool of oncology trained nurses and medical officers at primary care and district hospital level. These latter two phases are still outstanding. Figure 1 summarises the timeline of the current decentralisation process.

Evidently, the current decentralised model of care is only limited to patients requiring chemotherapy. It is hoped that the continued implementation of the decentralised model of cancer care will improve patient experience and quality of cancer care and reduce morbidity and mortality. However, data on patient demographic characteristics, epidemiological and clinical profile of various cancers is lacking in these two hospitals. This study therefore seeks to conduct an assessment of demographic, epidemiological and clinical profile of various cancers in NMAH and RFH. Furthermore, the study aims to describe the current process and

For peer review only - http://bmjopen.bmj.com/site/about/guidelines.xhtml

its benefits/challenges, with hopes of expanding 'decentralisation of care' in terms of services offered and number of decentralised hospitals.

Significance

South Africa's cancer services are generally urban-based and located in tertiary and quaternary health centres with an underdeveloped cancer service platform at district hospital and primary care levels. This means that patients needing cancer care have to travel long distances to big cities/towns in order to access basic cancer care. This creates gaps in access and quality of cancer care delivery between urban areas and rural areas. Decentralisation is a result of operational observations (to our knowledge there is no formal research that was done) such as long waiting times, delayed presentations, late diagnosis, patient complaints on travelling and out of pocket costs, etc. It is therefore envisaged that this study will provide insight on the distribution and types of cancers in areas where there is currently an underestimation of the burden of disease and as a result incorrect understanding of the levels of risk within the local populations. Moreover, establish the extent of the problem in both health facilities and possibly justify the need for decentralisation of cancer care services and help inform cancer preventive strategies in South Africa and other similar settings.

Objectives

- To describe the socio-demographic characteristics of patients diagnosed with cancer in the selected hospitals in the Eastern Cape and Mpumalanga provinces, South Africa.
- To determine and compare the incidence rate and prevalence of different types of cancer in the selected hospitals.
- To determine and compare the geographic distribution of cancers in the Eastern Cape and Mpumalanga provinces of South Africa.
- To determine the gaps between symptom development, first presentation at a health institution, first cancer diagnosis, referral for definitive management and initiation on treatment of patients diagnosed with cancer in South Africa's Eastern Cape and Mpumalanga provinces.
- To determine the comorbid conditions of patients with a cancer diagnosis in South Africa's Eastern Cape and Mpumalanga provinces.
- To determine the survival time of patients diagnosed with cancer in South Africa's Eastern Cape and Mpumalanga provinces.

For peer review only - http://bmjopen.bmj.com/site/about/guidelines.xhtml

METHODS AND ANALYSIS

Research design

This study will utilise a quantitative approach with a triangulation of a descriptive, exploratory cross-sectional and a longitudinal cohort design to answer the study objectives. The triangulation of designs compensates for the potential limitations of a single design. This study forms part of a bigger but yet to be published research project titled: “Exploring the feasibility, implications and outcomes of decentralising cancer care delivery in the Eastern Cape and Mpumalanga provinces of South Africa”.

Information will be sourced through two quantitative sub-studies, a cross-sectional survey with cancer patients and an ambi-directional cohort document review. Below is a brief description of the two sub-studies.

Sub-study 1: Quantitative cross-sectional study

A quantitative survey questionnaire will be administered on patients to assess demographic characteristics, prevalence of different cancers in selected hospitals and compare geographic distribution of cancers in the Eastern Cape and Mpumalanga provinces.

Sub-study 2: Quantitative ambi-directional cohort study design

Using the cancer patient registry (the registry is similar to a clinic logbook, but it is in an electronic form) used in the study sites, a document review will be carried out on cancer patients to ascertain the duration of cancer diagnosis, type of cancer(s), and the duration of survival since admission to the oncology clinic (survival time). Figure 2 shows the ambi-directional component of the study. Table 1 summarises the two sub-studies.

For peer review only

Table 1. Research methods summary

Sub-study	Study design	Objectives	Analysis
1	Cross-sectional study	- Describe socio-demographic characteristics of patients.<br>- Determine and compare the geographic distribution of cancers.<br>- Determine cancer disease progression.	• Frequency tables, percentages, and graphs to summarise categorical variables.<br>• Mean, standard deviation and range to summarise

For peer review only - http://bmjopen.bmj.com/site/about/guidelines.xhtml

		- Determine comorbid conditions of cancer patients.	normally distributed numerical variables; or Median and interquartile range to summarise skewed numerical variables.<br>• Chi-squared statistics or Fisher's exact test to compare categorical variables between groups.<br>• Parametric and/or non-parametric tests to compare numerical variables between groups.<br>• Kaplan-Meier survival estimates, for survival time.<br>• Hazard ratios for predictors of death.
2	Ambi-directional cohort study<br><br>(document review)	- Determine and compare the incidence rate and prevalence of different types of cancer in the selected hospitals.<br>- Determine the comorbid conditions of patients with a cancer diagnosis.<br>- Determine the survival time of patients diagnosed with cancer.	

Study setting

The study is located in two rural provinces with a high degree of under-development and marginalisation, namely Eastern Cape and Mpumalanga provinces in South Africa (8). Generally, Eastern Cape and Mpumalanga provinces are characterised by lack of the necessary infrastructure, resources, and expertise to provide quality, safe and accessible radiotherapy, chemotherapy, palliative care services and surgical oncology services. Patients from rural communities, who generally cannot afford private healthcare and are dependent on state health services for cancer care, are compelled to travel long distances to the urban-based tertiary or quaternary cancer care centres in order to access cancer care. The study will be conducted in two hospitals, NMAH in Mthatha, Eastern Cape province and RFH in Mbombela, Mpumalanga province. NMAH is one of ten central hospitals in South Africa and is the only one that is located in a rural area. This level of care is meant to be a quaternary level of care. RFH is a tertiary level of care hospital.

These are two hospitals in their respective provinces that refer their patients to seek quality cancer care in hospitals which are further away. At times, it takes patients up to 3-days of traveling when attending to their cancer care appointments. For example, cancer patients from Mpumalanga's RFH travel more than 400 kilometres to the country's capital city, Pretoria. While cancer patients from the Eastern Cape's NMAH travel more than 200 kilometres to East

For peer review only

For peer review only - http://bmjopen.bmj.com/site/about/guidelines.xhtml

London to access quality cancer care at an urban-based tertiary hospital. An anomaly, as NMAH is statutorily a level of care higher than a tertiary hospital.

Population and Sampling

Purposive sampling was used to select the study hospitals. The hospitals were selected based on their levels of care, gazetted specialist packages of care and concerns about the existing package of cancer care services, and because they are currently implementing a decentralised model of cancer care delivery. Furthermore, the two hospitals aim to establish centres of excellence in cancer care, a network of cancer care satellite sites at district hospital level and community-based cancer care services.

A triangulation of approaches will be used to select study participants from the two hospitals.

Sub-study 1: Quantitative cross-sectional study (patients)

Systematic random sampling of patients visiting the oncology clinics' outpatient's department will be conducted by approaching every 5th patient on the queue until the sampling size has been reached. A total combined sample size for the two hospitals will be calculated using the equation, $n = \frac{p(1-p)z^2}{d^2}$ for a one-sided 95% confidence interval and a 5% significance level (z=1.96). Because the proportion (p) of cancer patients who are seen in the respective hospitals is not known, this (p) will be set at 50% and the margin of error (d) will be set at 5%. This thus yields a total minimum sample size of 385. To factor for data entry errors a further 10% (39) will be added to yield a desired sample size of 424 participants for the two sites. Participants will then be recruited proportionally to yield a sample size of 212 patients per site.

Sub-study 2: Quantitative ambi-directional cohort (document review)

Information will be extracted from the patient registry (the registry is similar to a clinic logbook, but it is in an electronic form) to respond to the questions on the extraction tool. All patients under the care of the unit at any stage between the 01st of March 2018 and the 28th of February 2022 will be included.

Data collection

A multi-method approach to data collection will be adopted to get a comprehensive picture on cancer in the selected hospitals in terms of demographic distribution of cancer, socio-

For peer review only - http://bmjopen.bmj.com/site/about/guidelines.xhtml

economic characteristics, prevalence, duration of diagnosis, etc. This approach will also compensate for the potential limitations of a single data collection method and to triangulate the data as a means of checking the consistency of the study findings.

Sub-study 1: Quantitative cross-sectional study (patients)

The aim of this survey is to assess socioeconomic demographic characteristics of cancer patients, prevalence of different cancers in selected hospitals, and compare geographic distribution of the different cancers from the end-user's perspective. This sub-study will adopt and utilise a standardised and validated quantitative survey tool (Appendix A) to collect data from patients. The survey tool for patients has 51 questions developed through literature review and whose content validity was reviewed by three experts (one Occupational medicine specialist, a Public Health Medicine specialist, and an Oncologist). The questionnaire asks about the patient demographic profile and Epidemiological and clinical profile of various cancers. To test and ensure the effectiveness, the survey tool was also piloted amongst six patients in the two hospitals. Once the pilot study was done, all necessary adjustments were made to the data collection tool, thus ensuring that all questions will enhance the validity and reliability of the study findings. On clarity, there was 100% agreement among all three experts. On relevance only one of the three experts scored one question as irrelevant to result in an average score Content Validity Index of 0.99 (99%), which still renders the tool valid. Appendix C shows the experts' scoring in detail. This questionnaire has been translated into the local languages (isiXhosa, siSwati, and isiZulu) to accommodate participants who might not be comfortable with English.

Sub-study 2: Quantitative ambi-directional cohort study (document review)

Using a data extraction tool (Appendix B), a document review will be conducted in addition to the survey questionnaire. The main aim of the document review is to ascertain information which could not be captured or verified from the survey questionnaire, including, duration of cancer diagnosis, types of cancer, and survival time of cancer patients from the date of diagnosis.

Data management and analysis

Quantitative data analysis will include capturing survey data into Microsoft Excel Office 2016 and exporting the data into STATA version 17 (STATA Corp, College Station, Texas, USA) for analysis. Some descriptive and categorical data will be compared using frequencies, percentages, and graphs. Numerical data will be explored for normality using the Shapiro-Wilk test (9). If normally distributed the mean, range and standard deviation will be used. If not

For peer review only - http://bmjopen.bmj.com/site/about/guidelines.xhtml

normally distributed, then the median, and interquartile range (IQR) will be used. The Wilcoxon rank sum or an appropriate two-sample t-test will be used to compare the mean or median age of cancer patients by cancer type and between the two sites depending on the normality of the distribution of age and/or the equality of variances. A test for the equality of variances will be performed before use of the two-sample t-tests, if numerical variables are normally distributed. The two-sample t-test for independent samples will be carried out if variances are equal, and Satterthwaite's modified t-test used if the variances are not equal. The Chi-squared or Fisher's exact tests will be used depending on the value of the expected frequencies. If expected frequencies are <5 in binary comparisons or if any one cell of a larger comparison has an expected frequency of <1 or more than 20% of the cells of nominal categorical comparisons have an expected frequency of <5 then the Fisher's exact test will be used. Kaplan-Meier survival estimates will be used to determine the survival time. Hazard ratios will be used to determine the predictors of death. The level of statistical significance will be set at p-value ≤0.05. The 95% confidence interval will be used for the precision of estimates. Through triangulation, the analysis of the survey will seek to reproduce the cancer prevalence, epidemiological profiles and demographic characteristics determined in sub-study 1. These should be similar as determined by statistical methods (95% confidence interval and two-sample test of proportions).

Limitations

The study could be limited by the quality of data or poor information systems thus resulting in missing data. The assessment of the data at three time points will allow for the reduction of missing data as we will note suspicious entries and/or missing data and request for assistance with correction/filling of the missing data using the patient records or confirming from patients and/or family members. Missing data will be analysed using complete case analysis. The main outcome (cancer related deaths) could be limited by the absence of a mechanism linking to the death certificate, and/or autopsy. Every unspecified natural cause of death will therefore be considered to be related to the cancer as a direct or associated cause of death.

Patient and public involvement

The planning of the cancer service expansion involved community representatives through hospital boards in workshops and meetings. Patients will be informed of the study at all stages through consultations and public notices in the study sites.

Ethics and dissemination

For peer review only

For peer review only - http://bmjopen.bmj.com/site/about/guidelines.xhtml

Ethics approval was obtained from the Human Research Ethics Committees of the University of the Witwatersrand (M210211) and Walter Sisulu University, South Africa (040/2020). Site access approval has been obtained from the Provincial Health Research Committees of the Eastern Cape (EC_202010_012) and Mpumalanga (MP_202011_002) provinces respectively. The study will abide by the 4 ethical principles of autonomy, beneficence, non-maleficence, and justice.

Participants will be informed that their participation in this study is voluntary and that their confidentiality will be maintained throughout the study. Participants will also be assured that they are free to withdraw at any stage of the study and could opt-out of questions that they are not comfortable with. All identifying information will be removed. All electronic records will be accessed through a password encrypted database that only the principal investigator has access to. No direct incentives will be issued to participants. Before initiating the self-administered questionnaires, informed consent forms will be signed by all study participants. A waiver of consent has been attained for the document review. Information sheets and consent forms will be translated into relevant local languages (isiXhosa, Siswati and isiZulu). They will also be assured that data collected will be used only for the purposes of the study.

Findings of the study will be disseminated widely to all stakeholders, including participants; and will be used to inform both provincial and national strategies to expand and sustain provision of high-quality cancer screening, diagnosis, treatment, and palliative services, and promote community-based cancer care programmes. Results will be presented at annual partner meetings, national and international conferences. Results will also be published in open access peer-reviewed journals to facilitate broad access to findings.

Authors' contributions

WC conceived the research, sourced funding, engaged stakeholders, completed the first draft of the manuscript and jointly approved final draft. ORM edited and commented on versions of the manuscript and incorporated and addressed feedback from the co-authors. SAM edited versions of the manuscript, provided methodological strategy, validated the quantitative survey tool, and jointly approved final draft. ZJ is the content expert, edited and commented on versions of the manuscript. BS facilitated ethics and research access approvals, edited version of the manuscript. VE validated the quantitative survey tool and edited versions of the manuscript. DH, LG, NW, CZ, LB, SK, JN, SS, OG and AM edited versions of the manuscript.

Funding statement

For peer review only - http://bmjopen.bmj.com/site/about/guidelines.xhtml

This work was supported by the Bristol Myers Squibb Foundation grant number [1028].

Competing interests

None declared

For peer review only - http://bmjopen.bmj.com/site/about/guidelines.xhtml

REFERENCES

1. Fitzmaurice C, Abate D, Abbasi N et a. Global, Regional, and National Cancer Incidence, Mortality, Years of Life Lost, Years Lived With Disability, and Disability-Adjusted Life-Years for 29 Cancer Groups, 1990 to 2017: A Systematic Analysis for the Global Burden of Disease Study | Enhanced Rea. 2018.

2. World Health Organisation. Cancer factsheet Geneva. Cancer [Internet]. 2020 [cited 2022 Feb 21]. Available from: https://www.who.int/news-room/fact-sheets/detail/cancer

3. Bray F, Ferlay J, Soerjomataram I, Siegel RL, Torre LA, Jemal A. 394 CA: A Cancer Journal for Clinicians Global Cancer Statistics 2018: GLOBOCAN Estimates of Incidence and Mortality Worldwide for 36 Cancers in 185 Countries. CA CANCER J CLIN. 2018;68:394–424.

4. Ott JJ, Ullrich A, Mascarenhas M, Stevens GA. Global cancer incidence and mortality caused by behavior and infection. 2010 [cited 2022 Feb 21]; Available from: https://academic.oup.com/jpubhealth/article/33/2/223/1588120

5. Jha P. The hazards of smoking and the benefits of cessation: A critical summation of the epidemiological evidence in high-income countries. 2020; Available from: https://doi.org/10.7554/eLife.49979

6. National Department of Health. National Cancer Strategic Framework for South Africa 2017 - 2022. National Cancer Strategic Framework for South Africa 2017 - 2022. 2017.

7. International Agency for Cancer Research. Globocan South Africa Geneva. 2020;

8. Ngyende A. Statistical release (Revised). 2011 [cited 2022 Feb 22]; Available from: www.statssa.gov.za

9. Vetter TR. Fundamentals of Research Data and Variables: The Devil Is in the Details. Anesth Analg. 2017;125(4):1375–80.

For peer review only - http://bmjopen.bmj.com/site/about/guidelines.xhtml

Acknowledgements

The authors would like to thank the support received from patient representatives and officials of the participating hospitals and provinces for their assistance.

Patient consent for publication

Not required as this is a protocol. Patients to give consent before enrolment into the study.

Figure 1. Timeline of the decentralisation process.

Note. Ω = performed most surgical interventions even before decentralisation.

¶ = Performed this function before decentralisation.

◊ = No provision of Chemotherapy in the hospitals due to lack of lamina flow.

Figure 2. Ambi-directional cohort study for sub-study 2.

Note. Data collection will commence in January 2022 till the end of September 2022. Record review of patients diagnosed with cancer from 1 March 2018 will be done. Patients will be followed up for survival time either retrospectively (cancer related deaths between 1 March 2018 to January 2022), or prospectively (cancer related deaths from January 2022 until the study's end date, 30 September 2022).

For peer review only

For peer review only - http://bmjopen.bmj.com/site/about/guidelines.xhtml

389 # Appendices

390 ## Appendix A

391 Survey questionnaire (patients)

392 ## Appendix B

393 Document review template

394 ## Appendix C

395 Questionnaire validation

For peer review only

For peer review only - http://bmjopen.bmj.com/site/about/guidelines.xhtml

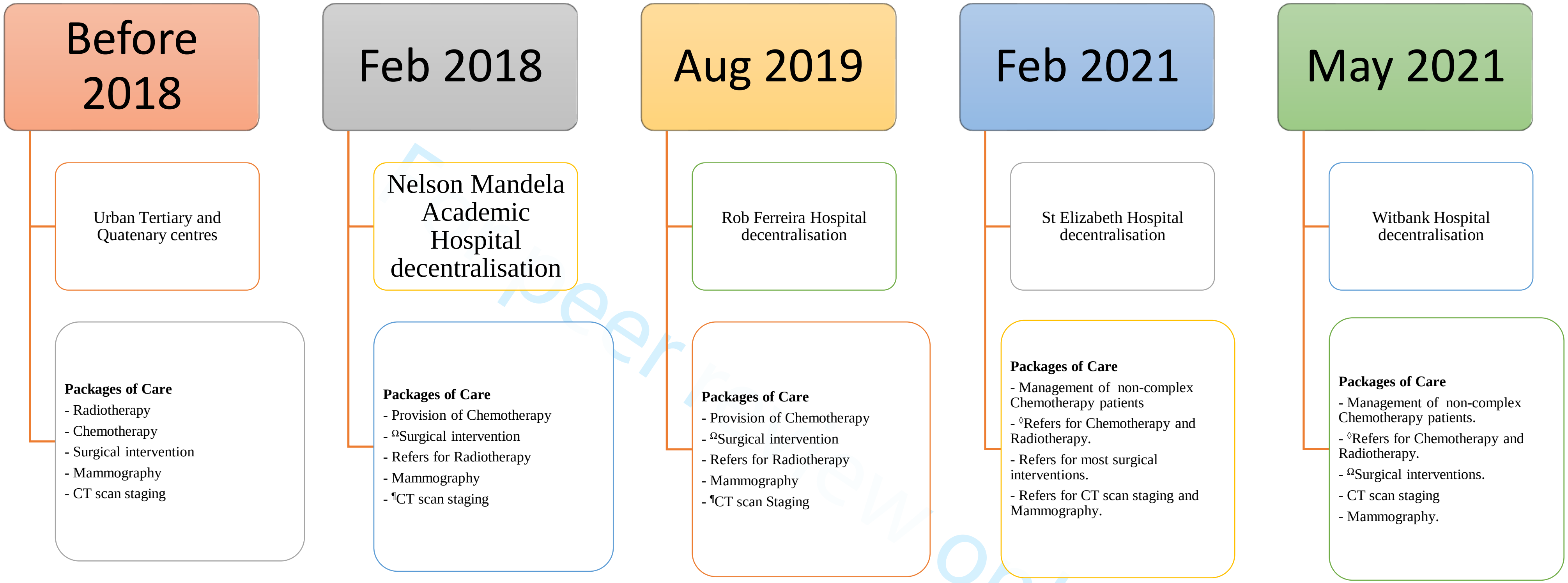

Note. Ω = performed most surgical interventions even before decentralisation.

¶ = Performed this function before decentralisation.

◊ = No provision of Chemotherapy in the hospitals due to lack of lamina flow.

For peer review only

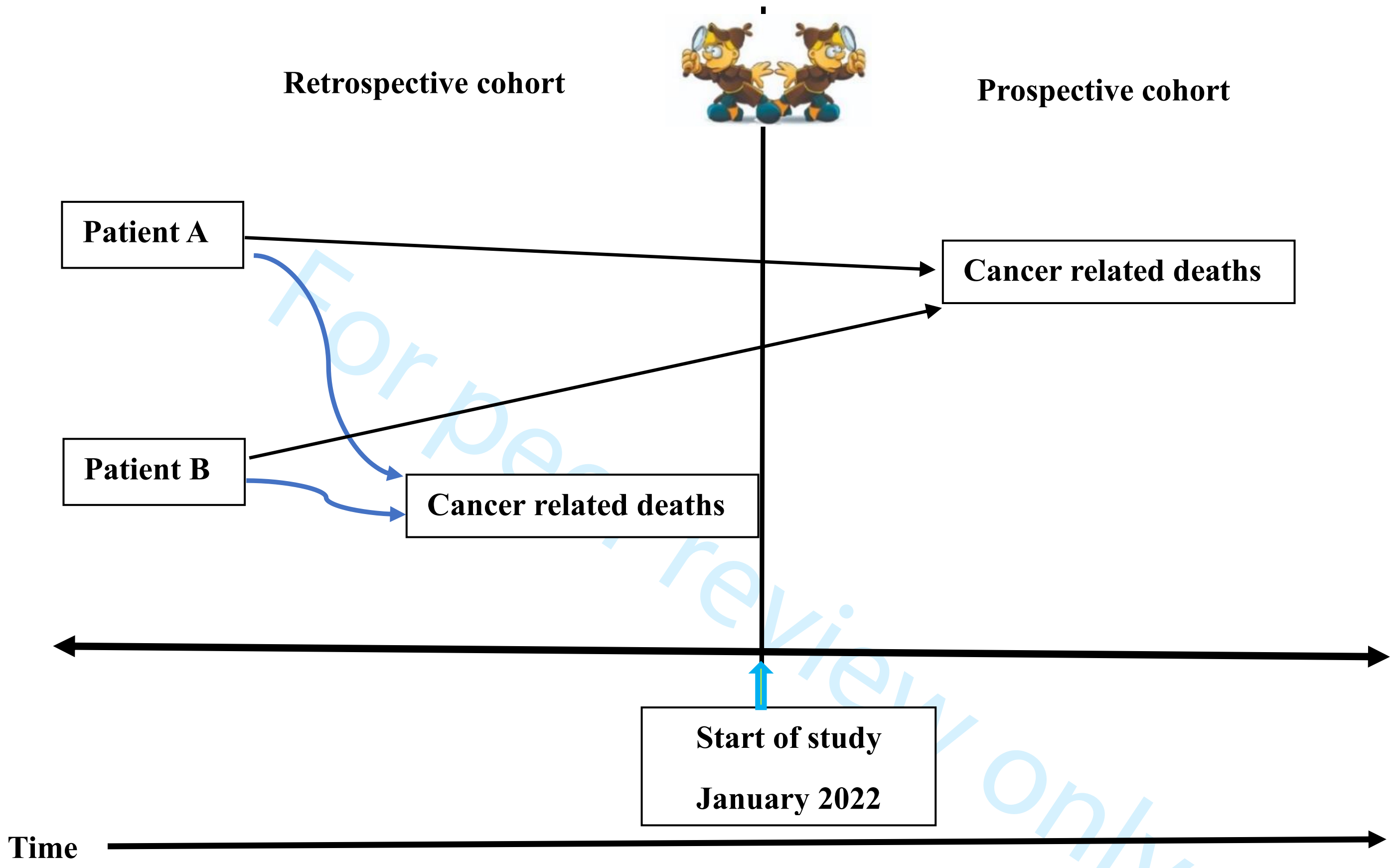

Note. Data collection will commence in January 2022 till the end of September 2022. Record review of patients diagnosed with cancer from 1 March 2019 will be done. Patients will be followed up for survival time either retrospectively (cancer related deaths between 1 March 2019 to January 2022), or prospectively (cancer related deaths from January 2022 until the study's end date, 30 September 2022).

Appendix A

Survey questionnaire (patients)

INSTRUCTIONS: Fill in the blank spaces with a tick where appropriate.

Date of Administration: ____________________________

Section 1: demographic profile

1. Gender

1	Female	
2	Male	

2. Date of Birth

Dd/Mm/Yy: | | | | | | | | |

3. Ethnicity

1	African	
2	White	
3	Indian	
4	Coloured	
5	Other: specify	

4. Marital status

1	Never Married	
2	Married (including lobola)	
3	Divorced/Separated	
4	Widowed	
5	Cohabiting	

5. What is the highest standard/grade or level you have passed at school or tertiary education?

For peer review only - http://bmjopen.bmj.com/site/about/guidelines.xhtml

For peer review only

6. Are you currently studying?

1	Yes	
2	No	

7. What is your current level of study? ---

8. Are you employed?

a)

1	Yes	
2	No	

b) If employed, what type of work do you do? ---

9. Source of income (tick all appropriate)

1	2	3	4	5	6	7	8	9
Job	Old Age Grant	Disability Grant	Other Pension	Spousal support	Support from children	Child support grant	None	Other (Specify)

10. What is your gross family income each month (that is, before tax)?

1	No income		7	R6 401 – R12 800	
2	R1 – R400		8	R12 801 – R25 600	
3	R401 – R800		9	R25 601 – R51 200	
4	R801 – R1 600		10	R51 201 – R102 400	
5	R1 601 – R3 200		11	R102 401 – R204 800	
6	R3 201 – R6 400		12	More than R204 800	

11. What is the name of your place of residence?____

12. Which health facility referred you here (Name)?

____

For peer review only - http://bmjopen.bmj.com/site/about/guidelines.xhtml

Section 2: Epidemiological and clinical profile of various cancers

13a. Do you have a family history of cancer?

1	2	3
Yes	No /	Unsure

13b. If yes to above, how are you related to the family member(s) who has the cancer diagnosis?

____
____

13c. What type of cancer(s) does the family member(s) have?

____

____

____

14. Do you smoke? (tick all appropriate)

a)

1	Yes	
2	No	

b) If Yes, when did you start smoking?

Year: __________

c) On average, how many cigarettes do you smoke in a day? ________________

d) If No, have you ever smoked?

1	Yes	
2	No	

e) For how long did you smoke? ______________

f) How many did you smoke in a day? _____________

1	Once	
2	Twice	
3	Three time	
4	More than 3 times	

For peer review only - http://bmjopen.bmj.com/site/about/guidelines.xhtml

For peer review only

g) Did you stop smoking?

1	Yes	
2	No	

h) How long ago did you stop smoking? ____

15. Do you drink alcohol?

a)

1	Yes	
2	No	

b) Did you drink alcohol before?

1	Yes	
2	No	

16. Do you exercise (physical) on a regular basis?

1	Yes	
2	No	

17. Have you ever worked in mines?

1	Yes	
2	No	

18. How long in years did you work in mines? --

19. Which mines (tick all that apply)? --

1	Gold	
2	Platinum	
3	diamond	
4	Coal	
5	Other (specify)	

For peer review only - http://bmjopen.bmj.com/site/about/guidelines.xhtml

For peer review only

20. Please indicate if your family has a history of any cancer/s below?

1	Breast cancer		6	Uterus cancer	
2	Lung cancer		7	Colon cancer	
3	Cervical cancer		8	Ovarian cancer	
4	Prostate Cancer		9	Other (specify)	
5	Oesophagus cancer		10	No history of cancer in my family	

21. Before you were told you needed to go to hospital about cancer, how many times did you see other doctors or health professionals about the health problem caused by cancer?

		1x	2x	3x	4x	5x	Other
1	I visited my local clinic						
2	I visited my local hospital						
3	I saw my local private doctor						
4	I saw my traditional healer/doctor/ Isangoma						
5	Other (specify):						

22. How do you feel about the length of time you had to wait before your first appointment with a hospital doctor or clinic doctor?

1	I was seen as soon as I thought was necessary	
2	I should have been seen a bit sooner	

23. How long was it from the time you identified symptoms?

----

24. Did your symptoms get better or worse or were the same while you were waiting for your first appointment with a hospital doctor?

----

25. What type of cancer(s) were you diagnosed with?

----

26. When was your cancer(s) diagnosed?

For peer review only - http://bmjopen.bmj.com/site/about/guidelines.xhtml

27. What health problems or symptoms did you notice at first?

----

28. Who first told you that you had cancer?

1	A hospital doctor	
2	A hospital nurse	
3	A GP (family doctor)	
4	Another health professional (specify)____________	
5	A friend or relative	
6	Nobody – I worked it out for myself	
7	Cannot remember	

29. When you were first told that you had cancer, had you been told you could bring a family member or friend with you?

1	Yes	
2	No	
3	It was not necessary	
4	I was told by phone or letter	
5	Don't know / Can't remember	
6	Other (specify)_________________	

30. How do you feel about the way you were told you had cancer?

----

30. Did you understand the explanation of what was found with you?

1	Yes	
2	No	

31. When you were told you had cancer, were you given written information about the type of cancer you had?

1	Yes	
2	No	

For peer review only - http://bmjopen.bmj.com/site/about/guidelines.xhtml

32. Before your cancer treatment started, were you given a choice of different types of treatment?

1	Yes	
2	No, but I would have liked a choice	
3	I was not given a choice because only one type of treatment was suitable for me	
4	Not sure / Can't remember	
5	Missing	

33. Do you think your views were taken into account when the team of doctors and nurses caring for you were discussing which treatment you should have?

1	Yes	
2	No	

34. Were the possible side effects of treatment(s) explained in a way you could understand?

1	Yes	
2	No	

35. Before you started your treatment, were you given verbal/written information about the side effects of treatment(s)?

1	Yes	
2	No	

36. Were you involved as much as you wanted to be in decisions about your care and treatment?

1	Yes	
2	No	

37a. During the last 12 months, have you had an operation (such as removal of a tumour or lump) at one of the hospitals named in the covering letter?

1	Yes	
2	No	
3	Not sure	

For peer review only - http://bmjopen.bmj.com/site/about/guidelines.xhtml

b. Before you had your operation, did a member of staff explain what would be done during the operation?

1	Yes	
2	No	

38. The last time you went into hospital for a cancer operation, was your admission date changed to a later date by the hospital?

1	Yes	
2	No	

39. Beforehand, were you given written/verbal information about your operation?

1	Yes	
2	No	

40. After the operation, did a member of staff explain how it had gone in a way you could understand?

1	Yes	
2	No	

41. As far as you know, was the hospital or your doctor that referred you for cancer treatment given enough information about your condition and the treatment you had at the hospital?

1	Yes	
2	No	
3	Don't know / Can't remember	

42. Do you think the doctors and nurses at your local hospital or clinic did everything they could to support you while you were in their care?

1	Yes	
2	No	

For peer review only

Appendix B

Document review template

##	Date of birth	Province	Site	Child	Patient Classification	Referred patient	Referral Type	Internal Referral Department	Referring Hospital_ Mpumalanga	- - -
1.										
2.										
3.										
4.										
5.										

##	Referring Hospital_EC	Referral Hospital	Gender	Race	Citizenship	Medical Aid	Postal code	Employed	Source of income	Specify Occupation	- - -
6.											
7.											
8.											
9.											
10.											

##	Previous work in mine	Number of Years worked in mines	Marital Status	Date of 1st oncology visit	Date of diagnosis	Cancer diagnosis 1	ICD10_Cancer diagnosis1	Cancer diagnosis1_ Stage	Cancer diagnosis 2	ICD10_Cancer diagnosis2	- - -
11.											
12.											
13.											
14.											
15.											

##	Cancer diagnosis2_Stage	Cancer diagnosis3	ICD10_Cancer diagnosis3	Cancer diagnosis3_ Stage	Chemotherapy	Onco_Drug1	Onco_Drug2	Neupogen	Hormonal Therapy	Blood Transfusion	- - -
16.											
17.											
18.											
19.											
20.											

##	Radiotherapy	PET_Bone Scan	Palliative Care	Social support	Psychological support	HIV	Hypertension	Diabetes Mellitus	COPD	Asthma	- - -
21.											
22.											
23.											
24.											
25.											

For peer review only

For peer review only - http://bmjopen.bmj.com/site/about/guidelines.xhtml

##	Other Chronic Disease1_Name	Other Chronic Disease2_Name	Family history of cancer	Previous Smoker	Current Smoker	Number of years of smoking	Number of cigarettes smoked in a day	Weight in Kg	Height in Centimetres	Date_of_ Current Visit1	---
26.											
27.											
28.											
29.											
30.											

##	Follow-up Date1	RIP Date	Date_of_Current Visit2	Follow-up Date2	---
31.					
32.					
33.					
34.					
35.					

For peer review only

For peer review only - http://bmjopen.bmj.com/site/about/guidelines.xhtml

Appendix C: Questionnaire validation

	**Relevance**						**Clarity**					
	Expert _1	Expert _2	Expert _3	Experts in agreement	Item Content Validity Index (I-CVI)	Universal Agreement (UA)	Expert _1	Expert _2	Expert _3	Experts in agreement	Item Content Validity Index (I-CVI)	Universal Agreement (UA)
**Survey questionnaire (patients) INSTRUCTIONS: Fill in the blank spaces with a tick where appropriate. Date of Administration:** _______________												
**Section 1: demographic profile**												
**Question**												
1. Gender<br>1 Female<br>2 Male	4	4	4	3	1	1	4	3	4	3	1	1
2. Date of Birth Dd/Mm/Yy: ________________	4	3	4	3	1	1	4	4	4	3	1	1
3. Ethnicity<br>1 African<br>2 White<br>3 Indian<br>4 Coloured<br>5 Other: specify	4	4	4	3	1	1	4	4	4	3	1	1
4. Marital status<br>1 Never Married<br>2 Married<br>3 Divorced/ Separated<br>4 Widowed<br>5 Cohabiting	3	3	4	3	1	1	4	3	4	3	1	1
5. What is the highest standard/grade you have attended in education? ------------	4	4	3	3	1	1	4	3	4	3	1	1
6. Are you currently studying?<br>1 Yes<br>2 No	3	3	3	3	1	1	4	3	3	3	1	1
7. What is your current level of study? --------------	3	3	3	3	1	1	4	3	4	3	1	1
8. Are you employed?<br>a)<br>1 Yes<br>2 No<br>b) If employed, what type of employment? ----------------------	4	4	4	3	1	1	4	4	4	3	1	1

For peer review only

9. Source of income (tick all appropriate)<br>1 Job<br>2 Old Age Grant<br>3 Disability Grant<br>4 Other Pension<br>5 Spousal support<br>6 Support from children<br>7 Child support grant<br>8 None<br>9 Other	4	4	4	3	1	1	4	4	4	3	1	1
10. What is your residential area? Name of town/administrative area: ----------	3	4	3	3	1	1	4	3	3	3	1	1
11. Referring facility:<br>1 Clinic<br>2 Community Health Centre<br>3 District Hospital<br>4 Regional Hospital<br>5 Private General practitioner<br>6 Private hospital<br>7 Other (specify):	4	4	4	3	1	1	4	4	4	3	1	1
**Section 2: Epidemiological and clinical profile of various cancers**				3	1	1				3	1	1
1. Do you have a family history of cancer?<br>1 Yes<br>2 No<br>3 Unsure	4	4	4	3	1	1	4	4	4	3	1	1
2. Do you smoke? (tick all appropriate)<br>1 Yes<br>2 No	4	4	4	3	1	1	4	3	4	3	1	1
2b) If Yes, when did you start smoking? Year: __________	4	3	4	3	1	1	4	3	3	3	1	1
2c) On average, how many cigarettes do you smoke in a day?	4	3	4	3	1	1	4	3	4	3	1	1
2d) If No, have you ever smoked?	4	3	4	3	1	1	4	3	4	3	1	1
2e) For how long did you smoke?	4	3	4	3	1	1	4	4	4	3	1	1

For peer review only

2f) How many did you smoke in a day?<br>1 Once<br>2 Twice<br>3 Three time<br>4 More than 3 times	4	3	4	3	1	1	4	3	3	3	1	1
2g) Did you stop smoking?<br>1 Yes<br>2 No	4	3	3	3	1	1	4	4	3	3	1	1
3. Do you drink alcohol?<br>a)<br>1 Yes<br>2 No	4	4	4	3	1	1	4	4	4	3	1	1
4. Do you exercise (physical) on a regular basis?<br>1 Yes<br>2 No	4	4	3	3	1	1	4	4	4	3	1	1
4b) Did you drink alcohol before?<br>1 Yes<br>2 No	4	4	4	3	1	1	4	4	4	3	1	1
5. Have you ever worked in mines?<br>1 Yes<br>2 No	4	4	4	3	1	1	4	3	4	3	1	1
6. How long in years did you work in mines? ------------	4	4	4	3	1	1	4	4	4	3	1	1
7. Which mines? Gold, diamond coal, mixed? --------	4	4	4	3	1	1	4	4	4	3	1	1
8.<br>a) Please indicate if your family has a history of any cancer/s below?<br>1 Breast cancer<br>5 Oesophagus cancer<br>2 Lung cancer<br>6 Colon cancer<br>3 Cervical cancer<br>7 Ovarian cancer<br>4 Prostate Cancer<br>8 Other (specify)<br>9 No history of cancer in my family<br><br>b) If you have history of cancer in your family, indicate who in the family had these cancer(s)?	4	4	4	3	1	1	4	4	4	3	1	1

For peer review only

9. Before you were told you needed to go to hospital about cancer, how many times did you see your GP (family doctor)/ clinic about the health problem caused by cancer?<br>1x 2x 3x 4x 5x Other<br>1 I visited my local clinic<br>2 I saw my local private doctor<br>3 I saw my traditional healer/doctor/ Isangoma<br>4 Other (specify):	4	4	4	3	1	1	4	4	4	3	1	1
10. How do you feel about the length of time you had to wait before your first appointment with a hospital doctor or clinic doctor?<br>1 I was seen as soon as I thought was necessary<br>2 I should have been seen a bit sooner	4	2	4	2	0,67	0	4	3	4	3	1	1
11. How long was it from the time you identified symptoms? -----------------------	4	4	4	3	1	1	3	4	3	3	1	1
12. Did your symptoms get better or worse or were the same while you were waiting for your first appointment with a hospital doctor? ----	4	4	4	3	1	1	4	4	4	3	1	1
13. What type of cancer(s) were you diagnosed with? ---	4	4	4	3	1	1	4	4	4	3	1	1
14. When was your cancer(s) diagnosed? ---------	4	4	4	3	1	1	4	4	4	3	1	1
15. What health problems or symptoms did you notice at first? ------	4	4	4	3	1	1	4	3	4	3	1	1

For peer review only

16. Who first told you that you had cancer?<br>1 A hospital doctor<br>2 A hospital nurse<br>3 A GP (family doctor)<br>4 Another health professional<br>5 A friend or relative<br>6 Nobody – I worked it out for myself<br>7 Cannot remember	3	4	4	3	1	1	4	4	4	3	1	1
17. When you were first told that you had cancer, had you been told you could bring a family member or friend with you?<br>1 Yes<br>2 No<br>3 It was not necessary<br>4 I was told by phone or letter<br>5 Don't know / Can't remember<br>6<br>Missing	3	4	4	3	1	1	3	4	4	3	1	1
18. How do you feel about the way you were told you had cancer? --------	4	4	4	3	1	1	4	4	4	3	1	1
19. Did you understand the explanation of what was found with you? --------	4	4	4	3	1	1	4	4	4	3	1	1
20. When you were told you had cancer, were you given written information about the type of cancer you had? -----	4	4	4	3	1	1	4	4	4	3	1	1
21. Before your cancer treatment started, were you given a choice of different types of treatment?<br>1 Yes<br>2 No, but I would have liked a choice<br>3 I was not given a choice because only one type of treatment was suitable for me<br>4 Not sure / Can't remember<br>5 Missing	4	4	4	3	1	1	4	4	4	3	1	1

For peer review only

22. Do you think your views were taken into account when the team of doctors and nurses caring for you were discussing which treatment you should have? ------	4	4	4	3	1	1	4	4	4	3	1	1
23. Were the possible side effects of treatment(s) explained in a way you could understand? --------	4	4	4	3	1	1	4	4	4	3	1	1
24. Before you started your treatment, were you given verbal/written information about the side effects of treatment(s)? -------	4	4	4	3	1	1	4	4	3	3	1	1
25. Were you involved as much as you wanted to be in decisions about your care and treatment? -----	4	4	4	3	1	1	4	4	4	3	1	1
26. a) During the last 12 months, have you had an operation (such as removal of a tumour or lump) at one of the hospitals named in the covering letter?<br>1 Yes<br>2 No<br>3 Not sure	4	4	4	3	1	1	4	4	4	3	1	1
26.b) Before you had your operation, did a member of staff explain what would be done during the operation? --------	4	4	4	3	1	1		4	4	3	1	1
27. The last time you went into hospital for a cancer operation, was your admission date changed to a later date by the hospital? -----------	4	4	4	3	1	1	4	4	4	3	1	1
28. Beforehand, were you given written/verbal information about your operation? ---	4	4	4	3	1	1	3	4	4	3	1	1

For peer review only

29. After the operation, did a member of staff explain how it had gone in a way you could understand? -------------	4	4	4	3	1	1	4	4	4	3	1	1
30. As far as you know, was your doctor given enough information about your condition and the treatment you had at the hospital?<br>1 Yes<br>2 No<br>3 Don't know / Can't remember	4	3	4	3	1	1	3	3	4	3	1	1
31. Do you think the doctors and nurses at your general practice/local clinic did everything they could to support you while you were at general practice or local clinic? ------	4	4	4	3	1	1	4	3	4	3	1	1
	**Total**				**50,67**	**50**					**51**	**51**
	Average Score-Content Validy Index (**S-CVI**) = I-CVI/n				**0,99352941**	**0,98**				**S-CVI**	**1**	**1**
	n = 51											
	**Proportion relevance**						**Proportion Clarity**					
	Expert 1	1					Expert 1	1				
	Expert 2	0,98					Expert 2	1				
	Expert 3	1					Expert 3	1				

**Likert Scale:**	
**Relevance**	**Clarity**
1 = Item is not relevant to the measured domain	1 = Item is not clear
2 = Item is somewhat relevant to the measured domain	2 = Item needs some revision
3 = Item is quiet relevant to the measured domain	3 = Item is clear but need some minor revision
4 = Item is highly relevant to the measured domain	4 = Item is very clea

For peer review only
